# Ionizing radiation-induced mutagenesis.

**DOI:** 10.1038/bjc.1988.2

**Published:** 1988-01

**Authors:** L. H. Breimer

**Affiliations:** Department of Chemical Pathology, Royal Postgraduate Medical School, London, UK.


Br. J. Cancer (1988), 57, 6–18 © The Macmillan Press Ltd., 1988

**REVIEW**

### Ionizing radiation-induced mutagenesis

L.H. Breimer*

*Department of Chemical Pathology, Royal Postgraduate Medical School, London W12 0HS, UK.*

Ionizing radiation was the first environmental agent shown to be mutagenic (Muller, 1927) and is now recognized as a pancarcinogen (Upton, 1984; Shigematsu & Kagan, 1986). Due to background irradiation and modern medicine it is, apart from oxygen, probably the most common carcinogenic agent to which humans are exposed. Ionizing radiation can cause neoplastic transformation of human cells in culture, but such transformation of cells in culture is not due to a single event (Borek, 1980; Kennedy *et al.*, 1980, 1984). Ionizing radiation exerts the bulk of its effects through the generation of oxygen-derived free radicals, in particular the hydroxyl radical (Teoule & Cadet, 1978; Hutchinson, 1985). Such reactive forms of oxygen also occur by chance as a byproduct of aerobic metabolism (Gerschman *et al.*, 1954; Di Guiseppi & Fridovich, 1984). It has been suggested that many dietary mutagens and carcinogens, tobacco smoke and other cancer promoters, act through the production of such radicals (Ames, 1983; Joenje, 1983; Cerutti, 1985) and the deleterious free radical reactions involving oxygen have also been implicated in the genesis of spontaneous cancer in man (Totter, 1980).

Although the literature on the genetic effects of ionizing radiation is immense, the molecular mechanism(s) involved are for technical reasons not as clearly understood as in the case of ultraviolet (UV) irradiation or alkylating agents. One reason is the large number of radiation products induced in DNA, possibly more than 100 (Teoule & Cadet, 1978; Hutchinson, 1985), so that correlating mutagenesis with a specific lesion is a formidable task. Another reason is that the mutagenic effect relative to the cell killing effect is less for ionizing radiation than for UV light or alkylating agents, and therefore is more difficult and, as a corollary, less appealing to study. The topic has been the subject of several reviews in the past 10 years (Hutterman *et al.*, 1978; Thacker & Cox, 1983; Hutchinson, 1985; Breimer & Lindahl, 1985*b*; Thacker 1985, 1986). Since Thacker's review (1986) was written, not only have the changes in DNA organization, as detected by Southern blotting, of mutants at several loci been reported, but also changes in DNA sequence. Here I review recent advances and attempt to evaluate ideas of a common mechanism of DNA damage and/or repair in spontaneous and ionizing radiation-induced mutation.

### Studies on the effect of ionizing radiation at specific genetic loci

The comparative importance of the base alterations induced by ionizing radiation in mutagenesis has been indicated by genetic experiments in bacteria, bacteriophages and lower eukaryotes (Glickman *et al.*, 1980; Levin *et al.*, 1982; Conkling *et al.*, 1976; Malling & de Serres, 1973; Das *et al.*, 1986). Base substitutions are the most common type of mutational event caused by ionizing radiation, both transitions and transversions being found at A.T as well as G.C base pairs. These base substitutions appear to be random (Glickman *et al.*, 1980; Kato *et al.*, 1985). However, these results have been obtained either by studying the reversion of nonsense mutations or by reverting radiation-induced mutants with point mutagens, and therefore there is some limitation to the types of events which can be detected (Schaaper *et al.*, 1986).

Tindall *et al.*, (1987) have studied the nature of a large number of ionizing radiation-induced forward mutations at the lambda *cI* locus. Irradiated phage was found to be mutated both when assayed in SOS (error prone repair) induced and non-induced host cells (recA deficient cells). This was in contrast to UV light which requires the induced SOS system, though the yield of mutants was higher than in the induced cells. The majority (~85%) of the mutants analysed were base-pair changes, the rest were frameshifts (though the analysis underestimates frameshifts). As mutations caused by double-strand breaks would not be detected in the phage experiments, they also studied the effect of irradiating lambda as prophage (i.e. already integrated in the *E. Coli* chromosome). The specificity of the base changes was lost though these still accounted for 78% of the mutation events. The proportion of frameshifts was unchanged but examples (6%) of internal rearrangements were also detected. The reason for these differences is not clear. The experimental conditions may favour base pair changes.

The role of base damage in ionizing radiation-induced mutagenesis in mammalian cells is unclear. A large volume of indirect evidence has been interpreted as suggesting that ionizing radiation does not induce simple base pair changes. This supposition is based on the observation that ionizing radiation does not increase the frequency of mutants at a locus coding for an essential gene where only point mutations are thought to be tolerated (ouabain resistance at the Na/K-ATPase locus) (Arlett *et al.*, 1975; Thacker *et al.*, 1978; Liber *et al.*, 1983). The frequency at the non-essential hypoxanthine-guanine phosphoribosyl transferase (*hprt*) locus, on the other hand, is significantly increased (Arlett *et al.*, 1975; Thacker & Cox, 1975; Liber *et al.*, 1983).

On the other hand, in a Chinese hamster ovary (CHO) cell line especially susceptible to point mutations, X-ray induction of diptheria toxin resistance can be observed (Wood *et al.*, 1983). Liber *et al.* (1986) have reported X-irradiation to induce mutations at two other essential, presumed 'point-mutation', loci. There has been one unconfirmed report of activation of a *ras* proto-oncogene by a radiation-induced single base alternation (G to A) (Guerrero *et al.*, 1984). However, as the *ras* system will only tolerate base changes (i.e. it is equivalent to reversion at a nonsense codon) and as the change recorded is a common spontaneous change, the significance of this finding is not clear.

Conclusive tests have been, until recently, hindered by an inability to characterize mutations in mammalian cells at the molecular level. However, the cloning of the genomic and cDNA sequences for specific genes such as adenine phosphoribosyl transferase (*aprt*) (Lowy *et al.*, 1980), hypoxanthine phosphorybosyl transferase (*hprt*) (Jolly *et al.*, 1982; Konecki *et al.*, 1982), dihydrofolate reductase (*dhfr*) (Chang *et al.*, 1978) and thymidine kinase (*tk*) (Bradshaw & Deininger, 1984) has made it possible to study directly the effects of mutagens on endogenous and exogenous genes.

*Present address: Institute of Cancer Research, Chester Beatty Laboratories, Fulham Road, London SW3 6JB, UK.
Received 2 July 1987; and in revised form, 9 October 1987.

Endogenous genes are genes which are part of the normal complement of the organism. Exogenous genes are foreign, often prokaryotic, DNA segments which have been introduced into the cell. Their position of integration and interaction with nuclear factors may affect their behaviour. The results of representative studies are summarized in Table I.

*Endogenous genes*

Most studies on the effect of ionizing radiation have been performed on the *hprt* locus in isolation. However, the study by Meuth's laboratory (Breimer *et al.*, 1986) characterized mutants at both *aprt* and *hprt* loci as well as determined changes in the DNA sequence. These studies show that there are locus specific effects influencing the type of mutation seen.

*hprt locus* The alterations in gene structure induced by ionizing radiation at the *hprt* locus have been determined at the level of restriction fragment length changes in Chinese hamster V79 cells (Vrieling *et al.*, 1985; Thacker, 1986; Fuscoe, *et al.*, 1986), CHO cells (Breimer *et al.*, 1986; Stankowski & Hsie, 1986; Gibbs *et al.*, 1987), and human lymphocytes (Skulimowski *et al.*, 1986; Liber *et al.*, 1987) (see Table I). The limit of resolution is ~500 bp. A major disadvantage of the *hprt* locus is that the gene is large (~39 kb) compared to the cDNA probe (1.3 kb), that is a probe of non-contiguous fragments is used, restricting the analysis.

About half of ionizing radiation-induced mutants in rodent cells had lost all detectable coding sequences. Another 20% were partial deletions. Breimer *et al.* (1986) also observed that the size of the *hprt* locus appeared to have increased, probably due to insertions, in the case of two mutants out of twelve examined. In these studies the vast majority (80–90%) of spontaneous mutations at the *hprt* locus showed no detectable change. A few partial deletions were observed; complete deletions were very rare (5%). The spectrum of changes induced by α-particles (Thacker, 1986) and those induced by decaying radioactive isotope ($^{125}I$) incorporated in the DNA (Gibbs *et al.*, 1987) were similar to those induced by γ-rays. Where both microscopic and molecular analysis have been performed together most of the chromosomal changes have been found to be unrelated to the location of the gene on the X-chromosome (Fuscoe *et al.*, 1986).

There were certain differences in the spectrum of changes observed in spontaneous and induced mutants of human cells in culture when compared with those seen in rodent cells (see Table I) (Skulimowski *et al.*, 1986; Liber *et al.*, 1987). In human cells there were relatively more radiation-induced mutants which showed no detectable change in gene organization and more spontaneous mutants which had totally lost the *hprt* gene than in rodent cells. The poor resolution of the *hprt* system and the lack of detailed description of the findings make it difficult to assess the relative importance of partial deletions, insertions and complex changes.

A molecular survey of *hprt* deficient patients showed that there was a marked genetic heterogeneity (~85% of cases appearing normal by DNA and RNA analysis) (Yang *et al.*, 1984; Wilson *et al.*, 1986).

**Table I** Frequency of changes in gene structure observed by Southern blot analysis in certain mutants in mammalian cells in culture arising spontaneously or induced by ionizing radiation (expressed as %)

| Mutant collection | No detectable alteration[a] | Only altered sites[b] | *Rearrangements* Partial deletions | Total deletions | Insertions | Complex |
|---|---|---|---|---|---|---|
| *Endogenous* | | | | | | |
| *aprt* | | | | | | |
| hamster[e] | | | | | | |
| spontaneous ($n=187$) | 88 | 7 | 4 | —[c] | 1 | 0 |
| ionizing radiation ($n=80$) | 81 | 1 | 8 | — | 1 | 9 |
| *hprt* | | | | | | |
| (i) hamster[f] | | | | | | |
| spontaneous ($n=52$) | 86 | — | 12 | 2 | 0 | 0 |
| ionizing radiation ($n=113$) | 31 | — | 27[d] | 41 | 2 | —[d] |
| (ii) human[g] | | | | | | |
| spontaneous ($n=28$) | 82 | — | 8 | 10 | 0 | 0 |
| ionizing radiation ($n=56$) | 43 | — | 7[d] | 30 | 20[d] | |
| *tk* | | | | | | |
| human[h] | | | | | | |
| normal growth | | | | | | |
| spontaneous ($n=51$) | 20 | — | — | 70 | 10 | |
| ionization radiation ($n=56$) | 32 | — | — | 63 | 5 | |
| slow growth | | | | | | |
| spontaneous ($n=120$) | 3 | — | — | 96 | 1 | |
| ionization radiation ($n=22$) | 4 | — | — | 91 | 4 | |
| *Exogenous* | | | | | | |
| *gpt* | | | | | | |
| hamster[k] | | | | | | |
| spontaneous ($n=23$) | 40 | 0 | 43 | 17 | 0 | 0 |
| ionizing radiation ($n=25$) | 0 | 0 | 4 | 96 | 0 | 0 |

[a]Limit of resolution; *aprt* approx. 25 bp; *hprt* approx. 500 bp; *tk* approx. 200 bp; [b]Loss or gain of restriction endonuclease cleavage site; [c]—; not reported; [d]*hprt*: some complex changes associated with partial deletions, not clearly reported. Data compiled from: [e]Nalbantoglu *et al.* (1983), Breimer *et al.* (1986), Grosovsky *et al.* (1986); [f]Fuscoe *et al.* (1983), Vrieling *et al.* (1985), Breimer *et al.* (1986), Fuscoe *et al.* (1986), Thacker (1986), Gibbs *et al.* (1987); [g]Skulimowski *et al.* (1986), Liber *et al.* (1987); [h]Yandell *et al.* (1986, 1987). The significance of the classification in 'normal growth' and 'slow growth' is not clear; [k]Stankowski & Hsie (1986).

*aprt locus* The endogenous genetic locus coding for the non-essential enzyme adenine phosphoribosyl transferase (*aprt*) in cultured Chinese hamster ovary cells is particularly attractive for analysis of mutational events because it offers a much greater resolution than any other mammalian locus used so far (Meuth & Arrand, 1982; Nalbantoglu *et al.*, 1983). The locus is small (3.9 kb) and contains a large number of restriction endonuclease recognition sites closely spaced, allowing detection and mapping of alterations as small as 25 bp. It has as low a spontaneous mutational rate as that of *hprt*. While the locus is autosomal, strains having only a single copy of the gene have been identified (Nalbantoglu *et al.*, 1983) facilitating the collection and analysis of a large number of mutants. The genomic sequence has now been determined (Nalbantoglu *et al.*, 1986*a*). Of nearly 200 spontaneous mutants, deletions amounted to ~4% and insertions <1% (see Table I). Other types of mutation (changes of <25 bp) were mapped to restriction sites in another 7% (Nalbantoglu *et al.*, 1983; Grosovsky *et al.*, 1986) and these are predominantly single base changes (Nalbantoglu *et al.*, 1987; de Jong *et al.*, 1987).

Breimer *et al.* (1986) characterized 25 independent mutants induced by ionizing radiation. In contrast to those at the *hprt* locus the pattern of *aprt* gene fragments of mutant DNAs were predominantly (75%) indistinguishable from the wild type pattern, suggesting that base pair changes or deletions/insertions <25 bp were responsible. One mutant had a grossly rearranged gene structure. Four mutants were deletions. These were small in size (one of 20 bp, two of 3.2 kb and one of 4 kb) as compared to several deletions >10 kb found among spontaneous *aprt* mutants (Nalbantoglu *et al.*, 1983, 1986*b*) and to those at *hprt* locus which eliminated all coding sequence detectable with a cDNA probe. Insertion mutations are rare at the *aprt* locus but one spontaneous and one γ-radiation-induced mutant have been identified and characterized.

The structure of the 20 bp γ-ray-induced deletion mutant was similar to that of spontaneously induced deletions in that it appeared to involve a non-homologous recombination event between a short direct repeat sequence, one copy of which was retained in the mutant gene (Breimer *et al.*, 1986; Nalbantoglu *et al.*, 1986*b*, 1987). (The DNA sequence of the larger deletion mutants caused by γ-irradiation has yet to be reported.)

The insertion mutations have also been sequenced (Breimer *et al.*, 1986; Meuth *et al.*, 1987). They are both small (58 bp in the case of the γ-ray-induced mutant, 285 bp for the spontaneous). They are accompanied by a deletion of 13 bp and 12 bp respectively at their sites of insertion. There is no duplication of flanking sequences. [Duplication of the flanking sequences at the position of insertion is a hallmark of insertion by transposable elements (Kleckner *et al.*, 1984; Weinert *et al.*, 1984; Grindley & Reed, 1985).] The target sites have some similarity but do not appear to be governed by homology with the inserted fragment. The sequences inserted in the spontaneous and radiation-induced mutants are very different. The insertion in the γ-irradiation-induced mutant has a sequence which is highly dispersed throughout the hamster genome, while the insertion in the spontaneous strain is unique. The hugely repetitive nature of the fragment inserted in the γ-radiation-induced mutant combined with the short direct repeats at one terminus and with the inverted repeats at the other end, makes it possible that it originally was longer and then was imprecisely excised.

Grosovsky *et al.* (1986) have also characterized genomic alterations in mutations at the *aprt* locus using the cell system developed by Meuth (see Table I). Likewise in their study only a few γ-ray induced and none of the spontaneously arising mutants had dectable genomic re-arrangements. Sixteen of the ionizing radiation-induced mutants showing no detectable change by Southern blot analysis have recently been sequenced (Grosovsky *et al.*, 1987). Eleven were due to single base changes. Every possible substitution event was observed, though the numbers were too few to determine any bias. The rest were deletions of frame shifts. Of the four deletions, two were similar in structure to those described by Meuth's group (Breimer *et al.*, 1986; Nalbantoglu *et al.*, 1986*b*). Three γ-ray-induced mutants were reported to have intragenic deletions, which may have similar breakpoints, but the significance of this is not clear. This study shows that ionizing radiation can cause mutations in mammalian cells through single base-pair changes, and that these may account for as many as half the mutations induced at the hamster *aprt* locus.

*dhfr and tk loci* Studies of genetic alterations at the dihydrofolate reductase (*dhfr*) locus in CHO cells detected deletions and rearrangements, though several mutants showed no detectable alterations of gene structure (Graf & Chasin, 1982). Unfortunately the resolution of this system is limited; in the cell line studied one of the copies of the autosomal *dhfr* locus was inactivated by a presumed point mutation. Thus the analysis was performed in a background of normal sized fragments, which precluded the detection of complete deletions of the gene. A hemizygous strain has since been developed (Urlaub *et al.*, 1983). With overlapping probes they can monitor a 210 kb region. The resolution is ~100 bp. Using this system Chasin's group (Mitchell *et al.*, 1986; Urlaub *et al.*, 1986) have analysed the changes in spontaneously arising and γ- and UV-irradiation-induced mutants. Of 5 spontaneous mutants none showed deletions. Three of these had lost exon V from their mRNA through a different single base change in the sequences critical for splicing. This area could be a mutational hot spot. Eleven γ-ray-induced mutants were also analysed. All showed major changes: there were 8 large deletions (3 of the full 210 kb) and 3 large inversions. The two deletion mutants induced by UV-irradiation were also large deletions (>95 and 210 kb respectively). Thus in this system ionizing radiation causes mutations through major genetic events. Surprisingly the deletions induced by UV-irradiation were as large, suggesting that when deletions occur at this locus they are generally substantial. Only two of the γ-ray-induced deletion mutants showed microscopically detectable changes in the region of the chromosome where the *dhfr* gene is located, but two of the three inversions had altered chromosome banding.

Little's group (Yandell *et al.*, 1986, 1987) have analysed a large number of mutations at the thymidine kinase (*tk*) locus in a human B-lymphoblastoid cell line that is heterozygous at the *tk* locus, with one functional and one non-functional allele (see Table I). The heterozygosity is thought to be due to a single base change because of the restriction enzyme site alterations, which also make it possible to distinguish the inactive copy from the active copy of the gene. The gene is located on chr 17, and they used informative molecular markers linked to the locus to detect large scale events. Among both induced and spontaneous mutants allele loss was more common (>70%) than intragenic mutation; in many cases, loss extended beyond the locus under selection, but only in a small fraction showed detectable changes in chr 17. The limit of resolution in this system is ~200 bp.

These studies on endogenous loci in mammalian cell lines in culture demonstrate that a significant number of mutations are caused by minimal changes in gene structure, a substantial proportion of which are point mutations. Previous reports (Cox *et al.*, 1977; Cox & Mason, 1978) that ionizing radiation mutations were caused by loss of, or microscopically visible damage to, the chromosome carrying the gene do not seem consistent with more detailed molecular analysis. Indeed, where both cytogenic and molecular analysis have been performed together, most of the chromosomal changes have been found to be unrelated to the known localization of the genes investigated (Fuscoe *et al.*, 1986; Urlaub *et al.*, 1986; Yandell *et al.*, 1987). There are also locus specific effects. Large deletions were the most common alterations induced by ionizing radiation at the *hprt*

and *dhfr* loci, whereas at the *aprt* locus the majority of mutants had alterations in gene structure of <25 bp and the deletions observed were small. The CHO cell lines in which the *aprt* and *dhfr* loci were studied are hemizygous for the locus, the other allele being totally deleted. Thus recombinational repair using the other allele is not possible, though this should favour gross errors rather than point mutations. In the case of the *hprt* locus the other X-chromosome is absent from the cell lines used. However, the X-chromosome is an unusual chromosome (e.g. it can undergo inactivation) and it is not known whether this affects the types of mutations induced. Nevertheless the *hprt* locus is not significantly more prone to spontaneous mutational events than the autosomal *aprt* locus. Another feature is that no deletions extending downstream of *aprt* have been isolated (Nalbantoglu *et al.*, 1983; Breimer *et al.*, 1986). This suggests that there is some essential function encoded there which will limit the types of mutations that can occur.

The studies of human cells give a different picture to that seen in rodent cells. At the *hprt* locus in human cells there were apparently more spontaneous mutants that had suffered total deletion of the gene than in the rodent cells. Total deletions increased less in number on $\gamma$-irradiation in human cells than in rodent cells and there were more radiation-induced mutants that had no detectable change in gene structure. At the endogenous *tk* locus little difference was seen between the two types of mutants, complete loss of the functional allele being common. Data from a mouse lymphoma cell line suggest that multilocus lesions are less likely to be lethal at the heterozygous *tk* locus than at the hemizygous *hprt* locus (Evans *et al.*, 1986), and analyses of the *aprt* locus in rodent cells as well as studies of certain human tumours support the notion that complex genetic events play a role in expression of recessive mutations at heterozygous loci (Koufos *et al.*, 1985; Cavanee, 1986). On the other hand, the finding that ionizing radiation but not mitomycin-C-induced mutations at the *tk* locus showed similar genetic changes to those of spontaneous mutants is reminiscent of the findings at the *aprt* locus, and could be interpreted that a similar molecular process is involved in the generation of either mutant. How the presence of a second copy of an allele rendered inactive by a presumed point mutation affects the repair of lesions in the expressed copy is not known. There is evidence that DNA base lesions are removed much more efficiently from active than inactive genes (Bohr *et al.*, 1985; Smith, 1987), suggesting that the cell has powers to discriminate. It is not clear whether cells could also use part of the inactive copy in repair. The mutational response to ionizing radiation is different for human and rodent cells, particularly at low doses (Thacker & Cox, 1975; Grosovsky & Little, 1985), though it has been suggested that these differences may be more related to differences in cell killing than mutagenesis (Thacker & Cox, 1975). Some caution is needed in the comparison of mutations between species as well as at dissimilar loci.

### *Exogenous genes and shuttle vectors*

As pointed out above among the difficulties of analysing changes at endogenous gene loci in mammalian cells are their size and complex structure. In addition there is now evidence that two genes can be encoded on opposite strands of the same DNA locus in mammalian cells (Adelman *et al.*, 1987) which could further limit the type of lesions tolerated. One way of circumventing these problems is to use a small, simple foreign gene either stably integrated in the gene line or as part of a 'shuttle-vector' system. In a shuttle-vector the gene studied is part of a construct which is able to replicate both in mammalian cells and another host, usually *E. coli*, so that the analysis is simplified (for technical details see Thacker, 1986). Early such vectors, especially those related to simian virus 40 (SV40), were prone to high levels of spontaneous mutagenesis (Razzaque *et al.*, 1983; Calos *et al.*, 1983; Hauser *et al.*, 1987). This has now been improved (Lebkowski *et al.*, 1985; Glazer *et al.*, 1986; MacGregor *et al.*, 1987). In particular the new vectors based on Epstein–Barr virus are likely to be of value in human cell systems (DuBridge *et al.*, 1987; Menck *et al.*, 1987). Unfortunately results of studies using shuttle vectors with ionizing radiation are not yet available.

Stankowski and Hsie (1986) have reported results of studies of mutations induced at the *E. coli gpt* gene in a CHO cell line (see Table I). The cell line was constructed from a *hprt* deletion mutant and contains a single, functional copy of the *gpt* stably integrated in its high molecular weight DNA. The gene is ~700 bp long and mutants were selected by thioguanine resistance. Nearly all of the mutants induced by known point mutagens showed no detectable changes in gene structure, but all those induced by ionizing radiation were deletions (all but one total) of the *gpt* gene. However, of the spontaneously arising mutations, most were partial or total deletions of the *gpt* gene; only 40% were unchanged. The results they reported for analysis of the *hprt* locus of a different CHO cell line were in agreement with those of other workers (see above). Though these data may be interpreted as evidence for ionizing radiation causing predominantly deletion mutations, the pattern of spontaneous mutations is different from that at *aprt* and *hprt* and closer to the situation at the endogenous *tk* locus. Whether this is due to some inherent property of the integrated prokaryotic DNA or the location of integration is not clear.

Goring *et al.* (1987) have analysed the spontaneous mutations in a chromosomally located single-copy Herpes simplex virus type I (HSV-I) thymidine kinase (*tk*) gene in a human $TK^-$ cell line. The *neo* gene, which codes for resistance to the antibiotic G418, was placed next to the *tk* gene for the purpose of screening out gross cheomosomal alterations. TK deficient mutants were selected with suitable drugs either separately or in combination. Analysis of mutations by Southern blotting revealed that the majority of mutants had undetectable (<50 bp) alterations of gene structure. Inactivation of the gene was not due to extensive methylation. A high reversion frequency suggested that point mutation might be the cause. These findings contrast with those at the endogenous *tk* locus (Yandell *et al.*, 1986, 1987). However, the few complex changes in gene structure, including total deletions, reported here were much more common when two drugs had been used to select mutants, suggesting that some of the undetectable alteration mutants could have been leaky or that the two agents used interact differently with the thymidine kinase enzyme. The close proximity of the *neo* gene will screen out larger deletions, and selecting for G418 resistance may affect the functional state of the genome in the region of the *neo* gene (Roginski *et al.*, 1983).

### DNA lesions and their repair

Ionizing radiation exerts the bulk of its damaging effects through oxygen derived free radicals, particularly hydroxyl radicals. It causes modification of the nitrogenous bases, single- and double-strand breaks and base-free (AP) sites (Ljungquist & Lindahl, 1974; Ward & Kuo, 1976; Cerutti, 1976; Teoule & Cadet, 1978; Hutchinson, 1985; Breimer & Lindahl, 1985*a*, *b*). For technical reasons most studies have been concerned with the formation and fate of strand breaks rather than base lesions. Indeed single-strand breaks have traditionally been regarded as the most frequent radiation lesion, but Hutchinson (1985) has estimated that base lesions are equally frequent, and Ward (1985) has calculated that base lesions may be at least twice as common as strand breaks.

### *Base damage*

*Pyrimidines* Ionizing radiation causes ring saturation, contraction and fragmentation of pyrimidines (Teoule *et al.*,

1977; Teoule & Cadet, 1978; Breimer & Lindahl, 1985*a*; Hutchinson, 1985). The action is mediated by attack by hydroxyl radicals at the 5,6 double bond and, in the case of thymine in DNA, can be mimicked by the oxidizing agents potassium permanganate ($KMnO_4$) and osmium tetroxide ($OsO_4$) though the spectrum of lesions is different (Breimer & Lindahl, 1980, 1984, 1985*a*). Briefly, thymine glycol (5,6-dihydroxy-6-hydrothymine) is the common lesion following $\gamma$-irradiation and oxidation. 5-Hydroxy-5-methylhydantoin, a minor $KMnO_4$ product, is readily formed by $\gamma$-irradiation. Methyltartronylurea, a major $KMnO_4$ product at physiological pH, is a minor $\gamma$-irradiation product. 6-Hydroxy-5,6-dihydrothymine is generated by $\gamma$-irradiation but not by oxidation; it is also a minor UV-photo product (Fisher & Johns, 1976). Urea, which is a potential end product of all base damage in DNA, is readily generated by $KMnO_4$ treatment, though its total yield is $\gamma$-irradiated DNA has not been unequivocally determined. The main additional thymine lesion is 5-hydroxymethyluracil ($\alpha$-hydroxythymine) (Teebor *et al.*, 1984). This base can substitute for thymine in the DNA of at least one bacteriophage without obviously altering the coding specificity.

Cytosine also undergoes ring reactions, but the glycol is unstable, and rupture is more common at the 4,5 bond. It is controversial whether ionizing radiation causes mutations through deamination of cytosine to uracil. Cytosine irradiated in solution can be deaminated to uracil, but this lesion has not been found in DNA (Teoule & Cadet, 1978; Hutchinson, 1985). Glickman *et al.* (1980) found no evidence that ionizing radiation increased mutations at 5-methylcytosine residues in *E. coli.* However, Tindall *et al.* (1987) report that two thirds of the mutations in irradiated lambda phage assayed in host cells not induced for the SOS system were G.C to A.T transitions and proposed that a cytosine product deaminated by irradiation was involved. An alternative explanation is that a non-deaminated product of cytosine, such as the ring contracted 1-carbamylimidazoldine-4,5-diol (Hahn *et al.*, 1973), could miscode as thymine and would not be generated from 5-methylcytosine.

The hydroxyl radicals initially generate unstable hydroxyhydroperoxidases of any base, which break down further into more stable derivatives. They may also be involved in forming protein DNA cross-links (Teebor *et al.*, 1984; Simic & Dizdaroglu, 1985). These hydroperoxides may be reconstituted to the original base through the action of glutathione dependent enzymatic processes thus limiting the damage (Tan *et al.*, 1986; Edgren, 1987).

The lesions induced in DNA are repaired by base excision through the action of DNA glycosylases, enzymes which cut the base-sugar bond of modified bases to release them in free form (Lindahl, 1982; Breimer & Lindahl, 1985*b*). In *E. coli* one single DNA glycosylase can remove several different forms of free radical altered thymine from DNA including thymine glycol, 5-hydroxy-5-methylhydantoin, methyltartronylurea and urea, and cleave the DNA at the resulting AP sites (Breimer & Lindahl, 1980, 1984). An analogous enzyme exists in mammalian, including human, cells. It has similar characteristics but is less active on thymine glycol than urea (Breimer, 1983; Hollstein *et al.*, 1984; Higgins *et al.*, 1987). It is also present in *Drosophila* (Breimer, 1986). 5-Hydroxymethyluracil-DNA glycosylase appears only to be present in differentiated mammalian cells (Hollstein *et al.*, 1984; Boorstein *et al.*, 1987). This is the first DNA glycosylase not to have a counterpart in lower organisms. Boorstein *et al.* (1987) have proposed that 5-hydroxymethyluracil in DNA may be weakly mutagenic because it slightly alters the physiochemical properties of DNA. To explain the phylogenetic difference they argue that unicellular organisms may tolerate a rare mutational event better than multicellular organisms, where supposedly even a very low mutagenic frequency could be disastrous. An alternative explanation would be that the altered base modifies coding by interacting with a mammalian protein or other factor not present or substantially different in lower organisms (i.e. that the presence of 5-hydroxymethyluracil in mammalian DNA causes mutation rather than these mutations being better tolerated in lower organisms). As even supplementing cell culture medium with thymidine can induce mutations through DNA precursor pool imbalance (Goncalves *et al.*, 1984; Phear *et al.*, 1987) this is unlikely to be a useful way of studying the properties of its 5-hydroxy derivative.

Several groups have now established that ring-saturated thymine lesions are non-coding rather than miscoding (Hariharan *et al.*, 1977; Ide *et al.*, 1985; Rouet & Essingmann, 1985; Clark & Beardsley, 1986; Hayes & Le Clerk, 1986). DNA templates have been oxidized to yield thymine glycols or further, unspecified, breakdown products (possibly urea). Essentially these experiments show that the thymine lesions strongly inhibit oxidized phage survival *in vivo* following transformation and DNA elongation by DNA polymerases *in vitro.* Sequence analysis showed that *in vitro* DNA synthesis terminated opposite thymine glycols but one nucleotide before putative urea residues. If the conditions of synthesis were relaxed by substitution of manganese for magnesium ions in the reaction mixture, dAMP was incorporated opposite the lesions. This is consistent with previous reports that DNA polymerases introduce dAMP residues opposite any non-informative lesion (Strauss *et al.*, 1982; Boiteux & Laval, 1982; Schaaper *et al.*, 1983; Kunkel, 1984) but it could also be due to thymine glycol coding as thymine (Ide *et al.*, 1985). The processivity was dependent on the presence of a pyrimidine preceding the thymine glycol. Virtually all these experiments have been done with single polymerase subunits or proteolytic fragments of the subunits, so they probably have relaxed fidelity of action. Moreover eukaryotic DNA polymerases are able to copy past non-informative lesions more readily than are enzymes purified from prokaryotes (Kunkel *et al.*, 1983).

No direct studies have been reported of the mutagenicity or repair of $\gamma$-irradiation-induced cytosine lesions. Though several are similar, they are not all equivalent to those of thymine.

*E. coli* mutants which are deficient in thymine glycol-DNA glycosylase ($nth^-$) are not more sensitive than wild type strains to the killing effects of ionizing radiation or hydrogen peroxide, though they show a weak mutator phenotype (Cunningham & Weiss, 1985). These results are not inconsistent with the DNA polymerase experiments. It has been reported that DNA exonuclease III (encoded by the *xth* gene) which accounts for 90% of total AP endonuclease activity in *E. coli* also can cut DNA at oxidized base residues other than thymine glycol (Kow & Wallace, 1985), which would enable the cell to deal with those lesions. However, *xth* defective mutants are extremely sensitive to hydrogen peroxide and ionizing radiation (Seeberg & Steinum, 1980; Demple *et al.*, 1982). The phenotype of $nth^- xth^-$ double mutants is not known. They may not be viable aerobically as they may be even more sensitive to oxygen free radicals.

*Purines* Ionizing radiation also causes saturation and fragmentation of the imidazole ring of purines (Teoule & Cadet, 1978; Bonicel, *et al.*, 1980; Breimer, 1984; Hutchinson, 1985; Cadet & Berger, 1985). Imidazole ring-opened adenine, 4,6-diamino-5-formamidopyrimidine, is structurally similar to imidazole ring-opened 7-methylguanine, which, if present in the template, blocks DNA chain elongation by *E. coli* DNA polymerase I *in vitro* (Boiteux & Laval, 1983). These ring-opened purines are excised from DNA by a DNA glycosylase, formamido-pyrimidine-DNA glycosylase (Chetsanga & Lindahl, 1979; Breimer, 1984) which is also present in mammalian cells (Breimer, 1983). The gene of the *E. coli* enzyme has now been cloned and sequenced (Boiteux *et al.*, 1987). Thus its physiological role should soon be known.

The C-8 position of purine can also be hydroxylated. Nishimura's group has shown that 8-hydroxy-deoxyguanosine (8-OH-dG) can be produced in DNA *in vitro* by various oxygen radical producing mutagenic or carcinogenic agents including ionizing radiation (Kasai *et al.*, 1984). 8-OH-dG was detected in DNA isolated from HeLa cells after cells in tissue culture had been irradiated with X-rays and from the liver of mice after the whole animal had been irradiated with $\gamma$-rays (Kasai *et al.*, 1986). The amounts of 8-OH-dG in DNA after *in vivo* irradiation were much lower than those after *in vitro* irradiation. The 8-OH-dG produced in liver DNA by irradiation of mice decreased with time, suggesting active repair.

Analysis of the effects of the 8-OH-dG residues in DNA on the fidelity of DNA replication using a DNA synthesis system *in vitro* with *E. coli* DNA polymerase I (Klenow fragment) showed that this lesion does not inhibit synthesis but miscodes (Kuchino *et al.*, 1987). In addition to misreading of the 8-OH-dG residue itself, pyrimidines next to the 8-OH-dG residue were also misread. When placed between T and C, the 8-OH-dG residue directed the insertion of A, T, C or G with an almost equal frequency, indicating that it lacks specific base-pairing. In addition to misreading of the 8-OH-dG residue itself, pyrimidines next to the 8-OH-dG residue (G was not tested) were also misread. When the DNA synthesis was carried out *in vitro* without addition of the dideoxynucleosidetriphosphates (ddNTP) used in the Sanger sequencing method almost no termination was detected. This newly synthesized DNA was analysed by the sequencing method of Maxam and Gilbert and misreading in the region of 8-OH-dG (in the absence of ddNTPs) confirmed. The reactions were performed under standard conditions and not 'relaxed', but again a fragment of DNA polymerase was used. Thus it is likely that 8-OH-dG residues in DNA will be directly mutagenic. Depending on its location it could induce two amino acid changes and therefore lead to mutations that would be extremely unlikely to revert and hence not appear as a single base damage event in indirect analyses. Moreover, Kasai *et al.* (1986) calculated that the extent of formation of 8-OH-dG in DNA by ionizing radiation in their experiments was of the same order as that of thymine glycol formation, previously thought to be the most frequent DNA base lesion. Studies using modified plasmids are now required to establish that 8-OH-dG is mutagenic *in vivo*. Such experiments have to be carefully planned, for it is now clear that if lesions are only present in one strand of a DNA molecule, there is specific strand loss in *E. coli* so that the undamaged strand alone is replicated (Koffel-Schwartz *et al.*, 1987).

Hypoxanthine, which is read as guanine, has been reported as a minor radiation product of adenine (Ponnamperuma *et al.*, 1961). It is excised by a DNA glycosylase (Karran & Lindahl, 1978, 1980). However, the spectrum of base changes reported so far do not indicate that it is a major mutagenic lesion of ionizing radiation.

*AP sites*

AP sites in DNA arise spontaneously through depurination (Lindahl & Nyberg, 1972). Ionizing radiation induces AP sites in DNA directly and as a result of the action of DNA glycosylases on damaged bases. By analysing the DNA from $\gamma$-irradiated HeLa cells Moran and Ebisuzaki (1987) have reported not only that DNA strand breaks are rapidly repaired but that AP sites were generated and subsequently repaired. The transient nature of the AP sites, reaching a maximum by 2 min, suggests that they are an early intermediate in a DNA repair pathway and that their removal may be rate limiting.

There is now considerable evidence that AP sites are mutagenic *in vivo* (Schaaper *et al.*, 1983; Kunkel, 1984; Miller & Low, 1984; Gentil *et al.*, 1984; Loeb, 1985; Granger-Schnarr, 1986; Cunningham *et al.*, 1986; Loeb & Preston, 1986; Foster & Davis, 1987). Analysis of DNA synthesis on templates containing AP sites has shown that dAMP residues are preferentially introduced opposite these lesions (Boiteux & Laval, 1982; Strauss *et al.*, 1982; Schaaper *et al.*, 1983; Kunkel, 1984). Such bypass can now also be achieved at AP sites under physiological conditions (Takeshita *et al.*, 1987). Thus ionizing radiation-induced AP sites will contribute to the mutational load. AP sites are subject to efficient repair by a number of DNA endonucleases (Ljungquist & Lindahl, 1974; Lindahl, 1979, 1982). Indeed the glycolase activity for radiation ring damaged thymine is also an endonuclease (Breimer & Lindahl, 1984 – see above). It has been reported that simultaneous presence of AP sites and base lesions in the same phage DNA interferes with the efficiency of their respective repair (Duker *et al.*, 1982). Tindall *et al.* (1987) reported a preferential substitution of A.T for any original base pair in irradiated lambda phage assayed in SOS induced hosts, and from Kunkel's work (1984), suggested that AP sites were the mutagenic intermediate. However, any non-informative lesion could in principle account for the result.

*Strand breaks*

DNA strand breaks are generated by a number of agents acting through oxygen derived free radicals, including ionizing radiation and radiomimetic drugs such as bleomycin. Single-strand breaks are generated directly by ionizing radiation, or as the result of the action of AP endonucleases with or without DNA glycosylases on lesions. Similarly double-strand breaks can be induced directly or as a result of enzymatic action at or near a single-strand break. Indeed, strand breaks are obligatory intermediates of excision repair, post replication repair and genetic recombination and any perturbation of their repair would be deleterious to the organism. The literature on ionizing radiation-induced strand-breaks and their physiological effects is enormous and confusing (for reviews see Cerutti, 1976; Hutterman *et al.*, 1978; Thacker & Cox, 1983; Hutchinson, 1985; Thacker, 1986). The initial number of strand-breaks is certainly a measure of the degree of insult (i.e. the total amount of energy deposited).

Briefly, $\gamma$-irradiation of DNA *in vitro* produces random single-strand breaks containing termini with 5′-phosphoryl-nucleotides on one side and on the other side either a normal nucleotide bearing a 3′-phosphoryl moiety or a 3′-phosphoryl glycolic acid ester formed from deoxyribose (Henner *et al.*, 1982, 1983; Hutchinson, 1985). Such single-strand interruptions are thus small gaps, resulting from the loss of at least one nucleoside. Bleomycin and neocarzinostatin, radiomimetic chemotherapeutic drugs, cause oxygen radical dependent strand breakage at thymines and generate complex termini (Giloni *et al.*, 1981; Kappen *et al.*, 1982).

The nature of the double-strand break directly arising from ionizing radiation is less well characterized but likely to be complex. Double-strand breaks *in vivo* increase linearly with dose, rather than the square of the dose, indicating the formation of each by a single event (Hutchinson, 1985). They are also affected differently by radiation conditions than single-strand breaks (Hutchinson, 1985).

Radiation-induced single-strand and double-strand breaks are repaired very rapidly in both prokaryotes and eukaryotes (Kapp & Smith, 1970; Karran & Ormerod, 1973; Resnick & Martin, 1976; Krasin & Hutchinson, 1977; Game *et al.*, 1980; Brenner *et al.*, 1986; Ayarez *et al.*, 1987). The repair of the double-strand breaks and some of the single-strand breaks evidently occur through a recombination mechanism. Recombinational repair permits the recovery of coding information lost as a result of the injury. It involves the donation of an intact DNA strand into the damaged duplex from a homologous sister or daughter chromosome (Szostak *et al.*, 1983). The repair of single-strand breaks and double-strand breaks appear to be complete even in cells irradiated at doses causing up to 99% killing (Lehman & Stevens, 1977; Hariharan *et al.*, 1981).

Substantial work has been undertaken to establish the effect of strand-breaks and their repair in mammalian cells (for review of techniques see Thacker, 1986). The results are generally inconclusive. To extrapolate from the results obtained by introducing into mammalian cells comparatively small (a few kb) segments of prokaryotic DNA which have been subjected to restriction enzyme digestion, to general models for *in vivo* repair of the highly organized mammalian cell DNA, is fraught with difficulties. Also, many of the vectors commonly used in these experiments, are prone to high levels of spontaneous mutagenesis in mammalian cells (see above). Also, some conclusions are based on introducing prokaryotic DNA endonucleases into eukaryotic cells. As the specificity of such enzymes can be affected by the reaction conditions, such data must be interpreted with caution.

There is indirect evidence that double-strand breaks may be the lesion responsible for chromosome aberration induced by ionizing radiation (for review see Thacker, 1986), though other groups have concluded that aberrations arise during the repair of induced DNA base damage (Preston, 1982). Recently a series of Chinese hamster ovary cell lines derived mutants (*xrs*) defective in the repair of double-strand breaks but containing normal levels of thymineglycol-DNA glycosylase have been isolated and characterized (Kemp *et al.*, 1984). The increased number of double-strand breaks remaining in these strains 20 min after irradiation, correlated well with the increase in chromosome breaks (Kemp & Jeggo, 1986).

Mammalian cells contain all the enzymes needed to mediate homologous recombination (Wake & Wilson, 1979; de Saint Vincent & Wahl, 1983). In prokaryotes and lower eukaryotes analysis of the mechanism and enzymology of recombination has been facilitated by the availability of mutants defective in this process often isolated because of radiation sensitivity (e.g. *rec* $A^-$ of *E. coli*). The *rad-52* mutants of yeast repair single-strand breaks but not double-strand breaks (Resnick & Martin, 1976) and are extremely deficient in both mitotic and meiotic recombination (Game *et al.*, 1980). Moore *et al.* (1986) have studied the recombination proficiency of the most extremely deficient double-strand break repair (*xrs*) mutant isolated by Kemp and Jeggo (1986). In an *in vivo* assay the mutants showed a 6-fold reduced homologous recombination frequency when compared to the parental cell line. However, *in vitro* assay of cell free extracts did not show any significant difference in proficiency. Hamilton and Thacker (1987) have further studied the *xrs* mutants and reported that while homologous recombination of plasmid molecules may not be substantially reduced in these *xrs* mutants, processes involved in the stable integration of plasmid DNA into genomic DNA are significantly impaired. Hoy *et al.* (1987) have reported that a CHO mutant which, though only slightly more sensitive to the effects of $\gamma$-irradiation, is considerably sensitive to some but not all alkylating agents and has a high level of sister chromatid exchange and decreased ability to rejoin strand breaks, is deficient in homologous recombination. Recently Jones *et al.* (1987) have reported the isolation of new X-ray sensitive mutants of V79-4 hamster cells. The availability of these mutants should help in characterizing DNA repair mechanisms.

### Cancer prone inherited disorders

A number of rare inherited disorders associated with increased sensitivity to ionizing radiation and risk of malignancy have been described, including ataxia telangiectasia (AT), Fanconi's anaemia (FA), Bloom's syndrome (BS) and inherited retinoblastoma. These syndromes could be regarded as naturally occurring mutants. There is a wealth of conflicting literature concerning these disorders partly because of extrapolation from data obtained with one or two cell lines to the whole syndrome. Cell free extracts of fibroblasts grown in culture failed to reveal deficiencies for AP endonucleases and urea-DNA glycosylase in the strains tested (Teebor & Duker, 1975; Breimer, 1983). Reports that AT fibroblasts were defective in excision repair of $\gamma$-ray damaged DNA (Paterson *et al.*, 1976) and that cell extracts of AT cells were deficient in an enzyme which enhanced the priming activity of $\gamma$-irradiated DNA for DNA polymerase I (Inoue *et al.*, 1977; Edwards *et al.*, 1980) have not been confirmed (van der Schans *et al.*, 1981; Shiloh *et al.*, 1981). The repair proficiency of these fibroblasts generally decrease with increasing passage (Sognier & Hittleman, 1983) making deficiency data difficult to interpret. Painter and Young (1980) have suggested that the cause of the increased radiation sensitivity in AT cells may be a defect in their ability to inhibit DNA synthesis following irradiation, thus giving less time for repair to take place. A single-strand break induced by ionizing radiation appears to be the stimulus for this effect; a break introduced by a repair activity has no effect (Painter & Young, 1987). There is evidence that AT cells are specifically sensitive to agents that break the deoxyribose moiety of DNA via a targetted free radical mechanism (Shiloh *et al.*, 1983; Joenje *et al.*, 1987). Cox *et al.* (1984, 1986) have proposed a model for the deficiency in AT. Normally there is a competition between ligation and exonuclease digestion of broken DNA. In AT cells a factor which modulates exonuclease activity is altered so that the balance is shifted from ligation to excessive digestion of DNA at the point of strand-breakage. The model is based on data derived from introducing vectors which are prone to spontaneous modification (see above) and the work awaits independent confirmation. There has been one unconfirmed report that the AT defect can be reversed by DNA transfection (Green *et al.*, 1987).

In whatever way DNA strand breaks arise and associated gaps are reconstituted, ultimately the DNA has to be joined together again, a reaction catalysed by DNA ligase (for review see Söderhall & Lindahl, 1976). Mammalian cells, in contrast to bacteria, have two different DNA ligases, designated I and II, both present in cell nuclei. They do not cross-react serologically. DNA ligase I, which is larger, is induced during cell proliferation and may be active during chromosomal replication, but ligase II is present in similar amounts in growing and nongrowing cells. Only ligase II can catalyse the joining of DNA–RNA hybrids (Arrand *et al.*, 1986). Using this differential assay Willis and Lindahl (1987) have shown that DNA ligase I is deficient in Bloom's syndrome. Independent data are consistent with this interpretation (Chan *et al.*, 1987).

In the case of inherited retinoblastoma it now appears that there is a somatic alteration of the normal allele such that it unmasks the mutant allele (Cavanee, 1986). In the sporadic form of the disease the tumorigenic version of the allele has to be acquired (a rare event), but in the familial form it is inherited.

### Possible future directions

In the last few years great advances have been made in analysing the molecular mechanisms of mutagenesis and carcinogenesis. It appears that locus specific effects are important in determining the types of mutations tolerated. There is now considerable evidence that ionizing radiation can induce mutations in mammalian cells through small genetic events, a substantial proportion of which are single base pair changes, rather than cytogenetically and microscopically observable events. However, the only way of ascertaining base changes in a gene (or the exact nature of any point mutation) is to determine the DNA sequence. For an endogenous gene this involves considerable labour of isolating, cloning and subcloning. The efficiency of the cloning procedure can be optimized by biological selection (Maniatis *et al.*, 1982; Grosovsky *et al.*, 1987). Nevertheless, even the *aprt* gene is too large to be sequenced conveniently and routinely.

A number of methods have been developed to increase the resolution of mapping the site of point mutations so that the DNA segment required to be sequenced can be limited. By careful optimization of the conditions of hybridization of the probe, chromosomal DNA from hundreds of bacterial mutants can potentially be examined for small changes including single base-pair changes without any DNA cloning (Miller & Barnes, 1986). The sensitivity of this method might be further increased by including tetramethylammonium chloride in the hybridization buffers, which abolishes the preferential melting at A.T *v* G.C base-pairs, thus making the procedure independent of the base composition of the probe (Wood *et al.*, 1985). Another method is the ribonuclease A cleavage procedure pioneered by Maniatis (Myers *et al.*, 1985). It is based on the property that some single base mismatches in RNA hybrids with RNA or DNA will be cleaved by RNase A, though the efficiency varies depending on the exact nature of the mismatch, certain base-pairs being cleaved poorly or not at all. Indeed mismatches resulting from deletions, insertions or rearrangements offer greater potential for RNase cleavage because of more extensive single-stranded regions within the hybrids. The method has been used by Gibbs and Caskey (1987) to identify and locate mutations at the *hprt* locus in Lesch-Nyhan patients not uncovered by other means. However, no such or any other mRNA analyses have been reported on ionizing radiation-induced mutants. Single base-pair mismatches can be detected in DNA by chemical modification which alters the migration of the DNA fragments during gel electrophoresis (Novack *et al.*, 1986). Single-stranded regions within a duplex fragment are accessible to carbodiimide, which reacts with unpaired G and T residues. Intact linear duplex DNA molecules do not react, whereas molecules containing single-base mismatches react quantitatively. After carbodiimide reaction the DNA molecules are electrophoresed in high percentage polyacrylamide gels to resolve modified and unmodified fragments. The resolution is best with small (<600 bp) fragments containing short single-stranded regions. However, the method could be made general by utilizing the repair enzyme UvrABC. It can cleave DNA at carbodiimide modified bases (E. Seeberg, personal communication; Thomas *et al.*, 1986). The enzyme cuts phosphodiester bonds four residues upstream and eight downstream of the lesion. Heteroduplexes are made between the wild type and mutated gene and treated with carbodiimide. The DNA is then cut on either side of the modified base by UvrABC. The resultant 12–13mer is released and the single-stranded region is cut with S1 nuclease. Thus two new fragments are generated. The fragments are then separated by electrophoresis on an agarose gel and detected and analysed in the usual manner. In principle there is no limit to the size of fragments that could be investigated.

However, the methods outlined above are still comparatively labour intensive. It is not clear whether the 'genomic sequencing' procedure described by Church and Gilbert (1984) can be used in mutation studies. An alternative would be to amplify the region of interest (Saiki *et al.*, 1985). Briefly, two oligonucleotide primers are synthesized that hybridize just beyond each end of the segment of interest, one to each DNA strand. The primers are annealed to denatured genomic DNA and then extended by DNA polymerase. Since the newly synthesized DNA strands are themselves templates for the primers, repeated cycles of denaturation, primer annealing, and extension results in exponential accumulation of the region defined by the primers (220,000-fold amplification has been described). It is not known how big a fragment can be amplified, nor whether the process is sufficiently specific, but the method could potentially dispense with the need of cloning and possibly allow analysis of mutants grown in microtitre plate wells.

A potentially powerful system would be to raise transgenic mice that contain a copy of a suitable shuttle vector in all cells of the animal. Such a strain could be used to perform mutagenesis experiments in the whole animal and could be of general use to approach physiologically relevant questions of mutagenesis.

The similarities observed between spontaneously arising and ionizing radiation-induced mutations at certain loci suggest that they may arise through common mechanisms of DNA change and/or repair. Ionizing radiation and other agents acting through oxygen free radicals may induce base changes through the generation of 8-hydroxylpurines in DNA, which miscode. Alternatively they may cause AP sites, either directly or as intermediates of repair. Likewise, the more complex changes of deletions and insertions could be due to similar initial lesions or compromised repair. Indeed, there is considerable evidence that the ability of cells to limit the effect of mutagenic insults may be of critical importance and that inadequate repair of lesions increases the rate of induction of mutations (Lindahl, 1982; Cunningham *et al.*, 1986; Smith, 1987; Foster & Davis, 1987; Yatagai *et al.*, 1987).

Some of the DNA-repair systems involved in the correction of alkylation damage and complex lesions are present in very small amounts intracellularly, but are inducible in *E. coli* (Witkin, 1976; Lindahl, 1982; Walker, 1984). Treatment with hydrogen peroxide induces an apparently analogous DNA repair pathway (Demple & Holbrook, 1983), and there is some overlap between functions induced and those induced through heat shock (Christman *et al.*, 1985; Privalle & Fridowitch, 1987). Paraquat, probably acting through oxygen-derived free radicals, can also induce DNA repair (Chan & Weiss, 1987). The exact mechanisms involved have yet to be established. Wolff's group has described an analogous mammalian response, in that incorporation of radioactive isotope into DNA or irradiation with a low dose induces resistance to a challenge dose of ionizing radiation (Oliveri *et al.*, 1984; Shadley & Wolff, 1987). Such induced cells could be sources of new repair enzymes, and defective mutants would establish their physiological importance.

### Conclusions

Data accumulated over the past two years demonstrate that ionizing radiation can induce mutations in mammalian cells through single base-pair changes at least at one locus. At other loci large scale events dominate. Microscopically visible chromosome changes play an insignificant part. The base-pair changes are probably mediated through miscoding base lesions such as 8-hydroxyguanine or AP sites. The similarity between some ionizing radiation-induced and spontaneously arising mutants is probably due to a common pathway of DNA damage (through oxygen-derived free radicals) and/or repair. However, the ultimate pattern of mutations will be established by analysing additional, different loci and by determining the DNA sequence of an adequate number of representative mutants. At present this is a formidable task, but with the development of more powerful methods of analysis and automated sequencing technology it is entirely feasible. The understanding of the cellular systems involved in limiting the effect of ionizing radiation will help in the design of more powerful radiosensitizers for clinical use and also in radioprotection.

I thank Drs B. Glickman, A. Grollman, A. Grosovsky, F. Hutchinson, J. Laval, J. Little, J. Nalbantoglu, B. Weiss and D. Yandell for communicating their manuscripts prior to publication. I also thank Drs P. Karran, T. Lindahl, S. West and R. Wood, and Profs A. Harris and I. MacIntyre for advice and encouragement. I thank Mrs B. Salvage for secretarial assistance. L.H.B. is a Fellow of the Beit Memorial Research Foundation.

### References

ADELMAN, J.P., BOND, C.T., DOUGLASS, J. & HERBERT, E. (1987). Two mammalian genes transcribed from opposite strands of the same DNA locus. *Science*, **235**, 1514.

AMES, B.N. (1983). Dietary carcinogens and anticarcinogens. Oxygen radicals and degenerative diseases. *Science*, **221**, 1256.

ARLETT, C.F., TURNBULL, D., HARCOURT, S.A., LEHMAN, A.R. & COLELLA, C.M. (1975). A comparison of the 8-azaguanine and ouabain resistance systems for the selection of induced Chinese hamster cells. *Mutat. Res.*, **33**, 261.

ARRAND, J.E., WILLIS, A.E., GOLDSMITH, I. & LINDAHL, T. (1986). Different substrate specificities of the two DNA ligases of mammalian cells. *J. Biol. Chem.*, **261**, 9079.

AYARES, D., GANEA, D., CHETURI, L., CAMPBELL, C.R. & KUCHERLAPATI, R. (1987). Repair of single-stranded DNA nicks, gaps and loops in mammalian cells. *Mol. Cell Biol.*, **7**, 1656.

BOHR, U.A., SMITH, C.A., OKUMOTO, D.S. & HANAWALT, P.C. (1985). DNA repair in an active gene: Removal of pyrimidine dimers from the DHFR gene of CHO cells is much more efficient than in the genome overall. *Cell*, **40**, 359.

BOITEUX, S. & LAVAL, J. (1982). Coding properties of poly(deoxycytidylic acid) templates containing uracil or apyrimidinic sites; *in vitro* modulation of mutagenesis by DNA repair enzymes. *Biochemistry*, **21**, 6746.

BOITEUX, S. & LAVAL, J. (1983). Imidazole open ring 7-methylguanine: An inhibitor of DNA synthesis. *Biochem. Biophys. Res. Commun.*, **110**, 552.

BOITEUX, S., O'CONNOR, T.R. & LAVAL, J. (1987). Formamidopyrimidine-DNA glycosylase of *E. coli*: Cloning and sequencing of the *fpg* structural gene and overproduction of the protein. *EMBO J.*, **6**, 3177.

BONICEL, A., MARIAGGI, N., HUGHES, E. & TEOULE, R. (1980). *In vitro* γ-irradiation of DNA: Identification of radio induced chemical modification of the adenine moiety. *Radiat. Res.*, **83**, 19.

BOORSTEIN, R.J., LEVY, D.D. & TEEBOR, G.W. (1987). 5-Hydroxymethyl-uracil-DNA glycosylase activity may be a differentiated mammalian function. *Mutat. Res.*, **183**, 257.

BOREK, C. (1980). X-ray induced *in vitro* neoplastic transformation of human diploid cells. *Nature*, **283**, 776.

BRADSHAW, H.D. & DEININGER, P.L. (1984). Human thymidine kinase gene: Molecular cloning and nucleotide sequence of a cDNA expressible in mammalian cells. *Mol. Cell Biol.*, **4**, 2316.

BREIMER, L.H. (1983). Urea-DNA glycosylase in mammalian cells. *Biochemistry*, **22**, 4192.

BREIMER, L.H. (1984). Enzymatic excision from γ-irradiated polydeoxyribonucleotides of adenine residues whose imidazole rings have been ruptured. *Nucleic Acids Res.*, **12**, 6359.

BREIMER, L.H. (1986). A DNA glycosylase for oxidized thymine residues in *Drosophila melanogaster*. *Biochem. Biophys. Res. Commun.*, **134**, 201.

BREIMER, L.H. & LINDAHL, T. (1980). A DNA glycosylase from *Escherichia coli* that releases free urea from a polydeoxyribonucleotide containing fragments of base residues. *Nucleic Acids Res.*, **8**, 6199.

BREIMER, L.H. & LINDAHL, T. (1984). DNA glycosylase activities for thymine residues damaged by ring saturation, fragmentation, or ring contraction are functions of endonuclease III in *Escherichia coli*. *J. Biol. Chem.*, **259**, 5543.

BREIMER, L.H. & LINDAHL, T. (1985*a*). Thymine lesions produced by ionizing radiation in double-stranded DNA. *Biochemistry*, **24**, 4018.

BREIMER, L.H. & LINDAHL, T. (1985*b*). Enzymatic excision of DNA bases damaged by exposure to ionizing radiation or oxidizing agents. *Mutat. Res.*, **150**, 85.

BREIMER, L.H., NALBANTOGLU, J. & MEUTH, M. (1986). Structure and sequence of mutations induced by ionizing radiation at selectable loci in Chinese hamster ovary cells. *J. Mol. Biol.*, **192**, 669.

BRENNER, D.A., SMIGOCKI, A.C. & CAMERINI-OTERO, R.D. (1986). Double strand gap repair results in homologous recombination in mouse L cells. *Proc. Natl Acad. Sci. USA.*, **83**, 1762.

CADET, J. & BERGER, M. (1985). Radiation-induced decomposition of the purine bases within DNA and related model compounds. *Int. J. Radiat. Biol.*, **47**, 127.

CALOS, M.P., LEBKOWSKI, J.S. & BOTCHAM, M.R. (1983). High mutation frequency in DNA transfected into mammalian cells. *Proc. Natl Acad. Sci. USA.*, **80**, 3015.

CAVANEE, W.K. (1986). The genetic basis of neoplasia: The retinoblastoma paradigm. *Trends in genetics*, **2**, 299.

CERUTTI, P.A. (1976). DNA base damage induced by ionizing radiation. In *Photochemistry and Photobiology of Nucleic Acids*, Wang, S.Y. (ed) vol. II, p. 375. Academic Press: New York.

CERUTTI, P.A. (1985). Prooxidant states and tumour promotion. *Science*, **227**, 375.

CHAN, E. & WEISS, B. (1987). Endonuclease IV of *E. coli* is induced by paraquat. *Proc. Natl Acad. Sci. USA.*, **84**, 3189.

CHAN, J.Y.H., BECKER, F.F., GERMAN, J. & RAY, J.H. (1987). Altered DNA ligase I activity in Bloom's syndrome cells. *Nature*, **325**, 357.

CHANG, A.C.Y., NUNBERG, J.H., KAUFMAN, R.J., ERLICH, H.A., SCHIMKE, R.T. & COHEN, S.N. (1978). Phenotypic expression in *E. coli* of a DNA sequence coding for mouse dehydrofolate reductase. *Nature*, **275**, 617.

CHRISTMAN, M.F., MORGAN, R.W., JACOBSON, F.S. & AMES, B.N. (1985). Positive control of a regulon for defence against oxidative stress and some heat-shock proteins in *Salmonella typhimurium*. *Cell*, **41**, 753.

CHETSANGA, C.J. & LINDAHL, T. (1979). Release of 7-methylguanine residues whose imidazole rings have been opened from damaged DNA by a DNA glycosylase from *Escherichia coli*. *Nucleic Acids Res.*, **6**, 3673.

CHURCH, G.M. & GILBERT, W. (1984). Genomic sequencing. *Proc. Natl Acad. Sci. USA.*, **81**, 1991.

CLARK, J.M. & BEARDSLEY, G.P. (1986). Thymine glycol lesions terminate chain elongation by DNA polymerase I *in vitro*. *Nucleic Acids Res.*, **14**, 737.

CONKLING, M.A., GRUNAU, J.A. & DRAKE, J.W. (1976). Gamma-ray mutagenesis in bacteriophage T4. *Genetics*, **82**, 565.

COX, R., THACKER, J., GOODHEAD, D.T. & MUNSON, R.J. (1977). Mutation and inactivation of mammalian cells by various ionising radiations. *Nature*, **267**, 425.

COX, R. & MASSON, W.K. (1978). Do radiation-induced thioguanine-resistant mutants of cultured mammalian cells arise by HGPRT gene mutation or X-chromosome rearrangement? *Nature*, **276**, 629.

COX, R., DEBENHAM, P.G., MASSON, W.K. & WEBB, M.B.T. (1986). Ataxia telangiectasia: A human mutation giving high frequency misrepair of DNA double strand scissions. *Molec. Biol. Med.*, **3**, 229.

COX, R., MASSON, W.K., DEBANHAM, P.G. & WEBB, M.B.T. (1984). The use of recombinant DNA plasmids for the determination of DNA repair and recombination in cultured mammalian cells. *Br. J. Cancer*, **49**, Suppl. VI, 67.

CUNNINGHAM, R.P. & WEISS, B. (1985). Endonuclease III (*nth*) mutants of *Escherichia coli*. *Proc. Natl Acad. Sci. USA.*, **82**, 474.

CUNNINGHAM, R.P., SAPORITO, S.M., SPITZER, S.G. & WEISS, B. (1986). Endonuclease IV (*nfo*) mutants of *Escherichia coli*. *J. Bacteriol.*, **168**, 1120.

DAS, G., STEWART, J.W. & SHERMAN, F. (1986). Mutational alterations induced in yeast by ionizing radiation. *Mutat. Res.*, **163**, 233.

DE JONG, P.J., GROSOVSKY, A.J. & GLICKMAN, B.W. (1987). Spectrum of spontaneous mutation at the *aprt* locus of CHO cells: An analysis at the DNA sequence level. *Proc. Natl Acad. Sci. USA.*, (in press).

DEMPLE, B.F. & HALBROOK, J. (1983). Inducible repair of oxidative DNA damage in *Escherichia coli*. *Nature*, **304**, 466.

DEMPLE, B., HALBROOK, J. & LINN, S. (1982). *Escherichia coli xth* mutants are hypersensitive to hydrogen peroxide. *J. Bacteriol.*, **153**, 1079.

DE SAINT VINCENT, B.R. & WAHL, G.M. (1983). Homologous recombination in mammalian cells mediates formation of a functional gene from two overlapping gene fragments. *Proc. Natl Acad. Sci. USA.*, **80**, 2002.

DIGUISEPPI, J. & FRIDOVICH, I. (1984). The toxicology of molecular oxygen. *C. R. C. Crit. Rev. Toxicol.*, **12**, 315.

DUBRIDGE, R.B., TANG, P., HSIA, H.C., LEONG, P.-M., MILLER, J.H. & CALOS, M.P. (1987). Analysis of mutation in human cells using an Epstein–Barr virus shuttle system. *Mol. Cell Biol.*, **7**, 379.

DUKER, N.J., JENSEN, D.E., HART, D.M. & FISHBEIN, D.E. (1982). Perturbations of enzymic uracil excision due to purine damage in DNA. *Proc. Natl Acad. Sci. USA.*, **79**, 4878.

EDGREN, M.R. (1987). Nuclear glutathione and oxygen enhancement of radiosensitivity. *Int. J. Radiat. Biol.*, **51**, 3.

EDWARDS, M.J., TAYLOR, A.M.R. & DUCKWORTH, G. (1980). An enzyme activity in normal and Ataxian Telangiectasia cell lines which is involved in the repair of $\gamma$-irradiation-induced DNA damage. *Biochem. J.*, **188**, 677.

EVANS, H.H., MENCK, J., HORNG, M.-F., RICEMTI, M., SANCHEZ, C. & HOZIER, J. (1986). Locus specificity in the mutability of the L5178Y mouse lymphoma cells: The role of multilocus lesions. *Proc. Natl Acad. Sci. USA.*, **83**, 4379.

FISHER, G.J. & JOHNS, H.E. (1976). Pyrimidine photohydrates. In *Photochemistry and Photobiology of Nucleic Acids*, Wang, S.Y. (ed) vol. I, p. 169. Academic Press: New York.

FOSTER, P.L. & DAVIS, E.F. (1987). Loss of an apurinic/apyrimidinic site endonuclease increases the mutagenicity of N-methyl-N'-nitro-N-nitrosoguanidine to *E. coli*. *Proc. Natl Acad. Sci. USA.*, **84**, 2891.

FUSCOE, J.C., FENWICK, R.G., LEDBETTER, D. & CASKEY, C.T. (1983). Deletion and amplification of the HPRT locus in Chinese hamster cells. *Mol. Cell Biol.*, **3**, 1086.

FUSCOE, J.C., OCKEY, C.H. & FOX, M. (1986). Molecular analysis of X-ray-induced mutants at the HPRT locus in V79 Chinese hamster cells. *Int. J. Radiat. Biol.*, **49**, 1011.

GAME, J.C., ZAMB, T.J., BRAUN, R.J., RESNICK, M.A. & ROTH, R.M. (1980). The role of radiation (*rad*) genes in meitoic recombination in yeast. *Genetics*, **94**, 51.

GENTIL, A., MARGOT, A. & SARASIN, A. (1984). Apurinic sites cause mutations in SV40. *Mutat. Res.*, **129**, 141.

GERSCHMAN, R., GILBERT, D.L., NYE, S.W., DWYER, P. & FENN, F.O. (1954). Oxygen poisoning and X-irradiation: A mechanism in common. *Science*, **119**, 623.

GIBBS, R.A. & CASKEY, C.T. (1987). Identification and localization of mutations at the Lesh–Nyhan locus by Ribonuclease A cleavage. *Science*, **236**, 303.

GIBBS, R.A., CARNAKARIS, J., HODGSON, G.S. & MARTIN, R.F. (1987). Molecular characteristics of $^{125}$I-decay and X-ray induced *hprt* mutants in CHO cells. *Int. J. Radiat. Biol.*, **51**, 193.

GILONI, L., TAKESHITA, M., JOHNSON, F., IDEN, C. & GROLLMAN, A.P. (1981). Bleomycin-induced strand-scission of DNA. *J. Biol. Chem.*, **256**, 8608.

GLAZER, P.M., SARKAR, S.N. & SUMMERS, W.C. (1986). Detection and analysis of UV-induced mutations in mammalian cell DNA using a $\lambda$-phage shuttle vector. *Proc. Natl Acad. Sci. USA.*, **83**, 1041.

GLICKMAN, B.W., RIETVELD, K. & ARON, C.S. (1980). $\gamma$-ray induced mutational spectrum in the *lac I* gene of *Escherichia coli*: Comparison of induced and spontaneous spectra at the molecular level. *Mutat. Res.*, **69**, 1.

GONCALVES, O., DROBETSKY, E. & MEUTH, M. (1984). Structural alterations of the *aprt* locus induced by deoxyribonucleoside triphosphate pool imbalances in Chinese hamster ovary cells. *Mol. Cell Biol.*, **4**, 1792.

GORING, D.R., GUPTA, K. & DuBOW, M.S. (1987). Analysis of spontaneous mutations in a chromosomally located HSV-1 thymidine kinase (TK) gene is a human cell line. *Somatic Cell & Mol. Genet.*, **13**, 47.

GRAF, L.H. & CHASIN, L.A. (1982). Direct demonstration of genetic alterations at the dihydrofolate reductase locus after gamma irradiation. *Mol. Cell Biol.*, **2**, 93.

GRANGER-SCHNARR, M. (1986). Base pair substitutions and frame shift mutagenesis induced by apurinic sites and two fluorene derivatives. *Mol. Gen. Genet.*, **202**, 90.

GREEN, M.H.L., LOWE, J.E., ARLETT, C.F. & 5 others (1987). A gamma-ray resistant derivative of an ataxia-telangiectasia cell line obtained following DNA-mediated gene transfer. *J. Cell Sci.*, Suppl. **6**, 127.

GRINDLEY, N.D.F. & REED, R.R. (1985). Transpositional recombination in prokaryotes. *Ann. Rev. Biochem.*, **54**, 863.

GROSOVSKY, A.J. & LITTLE, J.B. (1985). Evidence for linear responses for the induction of mutations in human cells by X-ray exposure below 10 rads. *Proc. Natl Acad. Sci. USA.*, **82**, 2092.

GROSOVSKY, A.J., DROBETSKY, E.A., DE JONG, P.J. & GLICKMAN, B.W. (1986). Southern analysis of genomic alterations in gamma-ray induced *aprt*$^-$ hamster cell mutants. *Genetics*, **113**, 405.

GROSOVSKY, A.J., DE BOER, J.G., DE JONG, P.J., DROBETSKY, E.A. & GLICKMAN, B.W. (1987). Base substitutions, frameshifts and small deletions comprise ionizing radiation induced point mutations in mammalian cells (submitted for publication).

GUERRERO, I., VILLASANTE, A., CORCES, V. & PELLICER, A. (1984). Activation of a c-K-*ras* oncogene by somatic mutation in mouse lymphomas induced by gamma radiation. *Science*, **225**, 1159.

HAHN, B.S., WANG, S.Y., FLIPPER, J.L. & KARLE, I.L. (1973). Radiation chemistry of nucleic acids. Isolation and characterisation of glycols of 1-carbamylimidazolidone as products of cytosine. *J. Amer. Chem. Soc.*, **95**, 2711.

HAMILTON, A.A. & THACKER, J. (1987). Gene recombination in X-ray sensitive hamster cells. *Mol. Cell Biol.*, **7**, 1409.

HARIHARAN, P.V., ACHEY, P.M. & CERUTTI, P.A. (1977). Biological effect of thymine ring saturation in coliphage X174-DNA. *Radiat. Res.*, **69**, 375.

HARIHARAN, P.V., ELECZKO, S., SMITH, B.P. & PATERSON, N.C. (1981). Normal rejoining of DNA strandbreaks in ataxia telangiectasis fiobroblasts after low X-ray exposure. *Radiat. Res.*, **86**, 589.

HAUSER, J., LEVINE, A.S. & DIXON, K. (1987). Unique pattern of point mutations arising after gene transfer into mammalian cells. *EMBO J.*, **6**, 63.

HAYES, R.C. & LE CLERK, J.E. (1986). Sequence dependence for bypass of thymine glycol in DNA by DNA polymerase I. *Nucleic Acids Res.*, **14**, 1045.

HENNER, W.D., GRUNBERG, S.M. & HASELTINE, W.A. (1982). Sites and structure of $\gamma$-irradiation-induced DNA strand breaks. *J. Biol. Chem.*, **257**, 11750.

HENNER, W.D., GRUNBERG, S.M. & HASELTINE, W.A. (1983). Enzyme action at 3′ termini of ionizing radiation induced DNA strand breaks. *J. Biol. Chem.*, **258**, 15198.

HIGGINS, S.A., FRENKEL, K., CUMMINGS, A. & TEEBOR, G.W. (1987). Definitive characterisation of human thymine glycol N-glycosylase activity. *Biochemistry*, **26**, 1683.

HOLLSTEIN, M.C., BROOKS, P., LINN, S. & AMES, B.N. (1984). Hydroxymethyluracil-DNA glycosylase in mammalian cells. *Proc. Natl Acad. Sci. USA.*, **81**, 4003.

HOY, C.A., FUSCOE, J.C. & THOMPSON, L.H. (1987). Recombination and ligation of transfected DNA in CHO mutant EM9, which has high levels of sister chromatid exchange. *Mol. Cell Biol.*, **7**, 2007.

HUTCHINSON, F. (1985). Chemical changes induced in DNA by ionizing radiation. *Progr. Nucleic Acid Res. Mol. Biol.*, **32**, 115.

HUTTERMANN, J., KOHNLEIN, W. & TEOULE, R. (1978). *Effects of Ionizing Radiation on DNA*, Springer-Verlag: Berlin.

IDE, H., KOW, Y.W. & WALLACE, S.S. (1985). Thymine glycols and urea residues in M13 DNA constitute replicative blocks *in vitro*. *Nucleic Acids Res.*, **13**, 8035.

INOUE, T., HIRANO, K., YOKOIYAMA, A., KADA, T. & KATO, H. (1977). DNA repair enzymes in ataxia telangiectasia and Bloom's syndrome fibroblasts. *Biochim. Biophys. Acta*, **479**, 497.

JOENJE, H. (1983). Oxygen: Our major carcinogen? *Med. Hypoth.*, **12**, 55.

JOENJE, H., NIEUWINT, A.W.M., TAYLOR, A.M.R. & HARNDEN, D.G. (1987). Oxygen toxicity and chromosomal breakage in ataxia telangiectasia. *Carcinogenesis*, **8**, 341.

JOLLY, D.J., ESTY, A.C., BERNARD, H.U. & FRIEDMANN, T. (1982). Isolation of a genomic clone partially encoding human hypoxanthine phosphoribosyltransferase. *Proc. Natl Acad. Sci. USA.*, **79**, 5038.

JONES, N.J., COX, R. & THACKER, J. (1987). Isolation and cross-sensitivity of X-ray-sensitive mutants of V79-4 hamster cells. *Mutant. Res.*, **183**, 279.

KAPP, D.S. & SMITH, K.C. (1970). Repair of radiation-induced strandbreaks in *E. coli*. II. Effect of *rec* and *uvr* mutations on radiosensitivity, and repair of X-ray induced single-stranded breaks in DNA. *J. Bacteriol.*, **103**, 49.

KAPPEN, L.S., GOLDBERG, I.H. & LIESCH, J.M. (1982). Identification of thymidine-5′-aldehyde at DNA strand breaks induced by neocarzinostatin chromophore. *Proc. Natl Acad. Sci. USA.*, **79**, 744.

KARRAN, P. & LINDAHL, T. (1978). Enzymatic excision of free hypoxanthine from polydeoxynucleotides and DNA containing deoxyinosine monophosphate residues. *J. Biol. Chem.*, **253**, 5877.

KARRAN, P. & LINDAHL, T. (1980). Hypoxanthine in DNA: Generation by heat-induced hydrolysis of adenine residues and release in free form by a DNA glycosylase from calf thymus. *Biochemistry*, **19**, 6005.

KARRAN, P. & ORMEROD, M.G. (1973). Is the ability to repair damage to DNA related to the proliferative capacity of a cell? The rejoining of X-ray-produced strandbreaks. *Biochim. Biophys. Acta*, **299**, 54.

KASAI, H., TANOOKA, H. & NISHIMURA, S. (1984). Formation of 8-hydroxyguanine residues in DNA by X-irradiation. *Gann*, **75**, 1037.

KASAI, H., CRAIN, P.F., KUCHINO, Y., NISHIMURA, S., OOTSUYAMA, A. & TANOOKA, H. (1986). Formation of 8-hydroxyguanine moiety in cellular DNA by agents producing oxygen radicals and evidence for its repair. *Carcinogenesis*, **7**, 1849.

KATO, T., ODA, Y. & GLICKMAN, B.W. (1985). Randomness of base substitution mutations induced in the *lac I* gene of *E. coli* by ionizing radiation. *Radiat. Res.*, **101**, 402.

KEMP, L.M. & JEGGO, P.A. (1986). Radiation-induced chromosome damage in X-ray-sensitive mutants (*xrs*) of the Chinese hamster ovary cell line. *Mutat. Res.*, **166**, 255.

KEMP, L.M., SEDGWICK, S.G. & JEGGO, P.A. (1984). X-ray sensitive mutants of Chinese hamster ovary cells defective in double-strand break rejoining. *Mutat. Res.*, **132**, 189.

KENNEDY, A.R., FOX, M., MURPHY, G. & LITTLE, J.B. (1980). Relationship between X-ray exposure and malignant transformation in CBH107$\frac{1}{2}$ cells. *Proc. Natl Acad. Sci. USA.*, **77**, 7262.

KENNEDY, A.R., CAIRNS, J. & LITTLE, J.B. (1984). Timing of the steps in transformation of C3H 10T1/2 cells by X-irradiation. *Nature*, **307**, 85.

KLECKNER, N., MORISATO, D., ROBERTS, D. & BENDER, J. (1984). Mechanism and regulation of Tn10 transposition. *Cold Spring Harbor Symp. Quant. Biol.*, **49**, 235.

KOFFEL-SCHWARTZ, N., MAENHAUT-MICHEL, G. & FUCHS, R.P.P. (1987). Specific strand loss in N-2-acetylaminofluorence modified DNA. *J. Mol. Biol.*, **193**, 651.

KONECKI, D.S., BRENNAND, J., FUSCOE, J.C., CASKEY, C.T. & CHINAULT, A.C. (1982). Hypoxanthine-guanine phosphoribosyltransferase genes of mouse and Chinese hamster: Construction and sequence analysis of cDNA recombinants. *Nucleic Acids Res.*, **10**, 6763.

KOUFOS, A., HAUSEN, M.F., COPELAND, N.G., JENKINS, N.A., LAMPKIN, B.C. & CAVANEE, W.K. (1985). Loss of heterogeneity in three enbryonic tumours suggests a common pathogenetic mechanism. *Nature*, **316**, 330.

KOW, Y.W. & WALLACE, S.S. (1985). Exonuclease III recognizes urea residues in oxidized DNA. *Proc. Natl Acad. Sci. USA.*, **82**, 8354.

KRASIN, F. & HUTCHINSON, F. (1977). Repair of DNA double-strand breaks in *Escherichia coli*, which requires *recA* function and the presence of a duplicate genome. *J. Mol. Biol.*, **116**, 81.

KUCHINO, Y., MORI, F., KASAI, H. & 5 others (1987). Misreading of DNA templates containing 8-hydroxydeoxyguanosine at the modified base and at adjacent residues. *Nature*, **327**, 77.

KUNKEL, T.A. (1984). Mutational specificity of depurination. *Proc. Natl Acad. Sci. USA.*, **81**, 1491.

KUNKEL, T.A., SCHAAPER, R.M. & LOEB, L.A. (1983). Depurination-induced infidelity of deoxyribonucleic acid synthesis with purified deoxyribonucleic acid replication proteins *in vitro*. *Biochemistry*, **22**, 2378.

LEBKOWSKI, J.S., CLANCY, S., MILLER, J.H. & CALOS, M.P. (1985). The *lac I* shuttle: Rapid analysis of the mutagenic specificity of UV-light in human cells. *Proc. Natl Acad. Sci. USA.*, **82**, 8606.

LEHMANN, A.R. & STEVENS, S. (1977). The production and repair of double-strand breaks in cells from normal humans and from patients with ataxia telangiectasia. *Biochim. Biophys. Acta*, **474**, 49.

LEVIN, D.E., HOLLSTEIN, M., CHRISTMAN, M.F., SCHWIERS, E.A. & AMES, B.N. (1982). A new Salmonella tester strain (TA 102) with A.T. base pairs at the site of mutation detects oxidative mutagens. *Proc. Natl Acad. Sci. USA.*, **79**, 7445.

LIBER, H.L., LE MOTTE, P.K. & LITTLE, J.B. (1983). Toxicity and mutagenicity of X-rays and [$^{125}$I]dUrd or [$^{3}$H]TdR incorporated in the DNA of human lymphoblast cells. *Mutat. Res.*, **111**, 387.

LIBER, H.L., LEONG, P.-M., TERRY, V.H. & LITTLE, J.B. (1986). X-rays mutate human lymphoblast cells at genetic loci that should only respond to point mutagens. *Mutat. Res.*, **163**, 91.

LIBER, H.L., CALL, K.M. & LITTLE, J.B. (1987). Molecular and biochemical analyses of spontaneous and X-ray-induced mutants in human lymphoblastoid cells. *Mutat. Res.*, **178**, 143.

LINDAHL, T. (1979). DNA glycosylases, endonucleases for apurinic/apyrymidinic sites, and base excision repair. *Prog. Nucleic Acid Res. Mol. Biol.*, **22**, 135.

LINDAHL, T. (1982). DNA repair enzymes. *Ann. Rev. Biochemistry*, **51**, 61.

LINDAHL, T. & NYBERG, B. (1972). Rate of depurination of native deoxyribonucleic acid. *Biochemistry*, **11**, 3610.

LJUNGQUIST, S. & LINDAHL, T. (1974). A mammalian endonuclease specific for apurinic sites in double-stranded deoxyribonucleic acid. *J. Biol. Chem.*, **249**, 1536.

LOEB, L.A. (1985). Apurinic sites as mutagenic intermediates. *Cell*, **40**, 483.

LOEB, L.A. & PRESTON, B.D. (1986). Mutagenesis by apurinic/apyrimidinic sites. *Ann. Rev. Genet.*, **20**, 201.

LOWY, I., PELLICER, A., JACKSON, J.F., SIM, G.-K., SILVERSTEIN, S. & AXEL, R. (1980). Isolation of transforming DNA: Cloning the hamster aprt gene. *Cell*, **22**, 817.

MACGREGOR, G.R., JAMES, M.R., ARLETT, C.F. & BURKE, J.F. (1987). Analysis of mutations occurring during replication of a SV40 shuttle vector in mammalian cells. *Mutat. Res.*, **183**, 273.

MALLING, H.V. & DE SERRES, F.J. (1973). Genetic alterations at the molecular level in X-ray induced *ad-3B* mutants of *Neurospora crassa*. *Radiat. Res.*, **53**, 77.

MANIATIS, T., FRITSCH, E.F. & SAMBROOK, J. (1982). *Molecular Cloning: A laboratory manual*, Cold Spring Harbor Laboratory, Cold Spring Harbor, New York.

MENCK, C.F.M., JAMES, M.R., GENTIL, A. & SARASIM, A. (1987). Strategies to analyse mutagenesis in mammalian cells using SV40 or shuttle vectors. *J. Cell Sci.*, Suppl. **6**, 323.

MEUTH, M. & ARRAND, J.E. (1982). Alterations of gene structures in EMS-induced mutants of mammalian cells. *Mol. Cell Biol.*, **2**, 1459.

MEUTH, M., NALBANTOGLU, J., PHEAR, G. & MILES, C. (1987). Banbury Report no. 28, *Mammalian Mutagenesis*, in press.

MEUTH, M., NALBANTOGLU, J., PHEAR, G. & MILES, C. (1987). Molecular basis of aprt gene rearrangements. In *Mammalian Cell Mutagenesis*, Moore, M.M., de Marini, D.M., de Serres, F. & Tindall, K.R. (eds) in press. Cold Spring Harbor Laboratory, New York.

MILLER, J.H. & LOW, K.B. (1984). Specificity of mutagenesis resulting from the induction of the SOS system in the absence of mutagenic treatment. *Cell*, **37**, 675.

MILLER, J.K. & BARNES, W.M. (1986). Colony probing as an alternative to standard sequencing as a means of direct analysis of chromosomal DNA to determine the spectrum ofd single-base changes in regions of known sequence. *Proc. Natl Acad. Sci. USA.*, **83**, 1026.

MITCHELL, P., URLAUB, G. & CHASIN, L. (1986). Spontaneous splicing mutations at the dihydrofolate reductase locus in Chinese hamster ovary cells. *Mol. Cell Biol.*, **6**, 1926.

MOORE, P.D., SONG, K.-Y., CHEKURI, L., WALLACE, L. & KUCHERLAPATI, R.S. (1986). Homologous recombination in a Chinese hamster X-ray-sensitive mutant. *Mutat. Res.*, **160**, 149.

MORAN, M.F. & EBISUZAKI, K. (1987). Base excision repair of DNA in $\gamma$-irradiated human cells. *Carcinogenesis*, **8**, 607.

MULLER, H.J. (1927). Artificial transmutation of the gene. *Science*, **66**, 84.

MYERS, R.M., LARIN, Z. & MANIATIS, T. (1985). Detection of single base substitutions by ribonuclease cleavage at mismatches in RNA:DNA duplexes. *Science*, **230**, 1242.

NALBANTOGLU, J., GONCALVES, O. & MEUTH, M. (1983). Structure of mutant alleles at the *aprt* locus of Chinese hamster ovary cells. *J. Mol. Biol.*, **167**, 575.

NALBANTOGLU, J., PHEAR, G.A. & MEUTH, M. (1986*a*). DNA sequence of Chinese hamster *aprt* gene. *Nucleic Acids Res.*, **14**, 1914.

NALBANTOGLU, J., HARTLEY, D., PHEAR, G., TEAR, G. & MEUTH, M. (1986*b*). Spontaneous deletion formation at the *aprt* locus of hamster cells: The presence of short sequence homologies and dyad symmetries at deletion termini. *EMBO J.*, **5**, 1199.

NALBANTOGLU, J., PHEAR, G. & MEUTH, M. (1987). DNA sequence analysis of spontaneous mutations at the *aprt* locus of hamster cells. *Mol. Cell Biol.*, **7**, 1445.

NOVACK, D.F., CASNA, N.J., FISCHER, S.G. & FORD, J.P. (1986). Detection of single base-pair mismatches in DNA by chemical modification followed by electrophoresis in 15% polyacrylamide gel. *Proc. Natl Acad. Sci. USA.*, **83**, 586.

OLIVERI, G., BODYCOTE, J. & WOLFF, S. (1984). Adaptive response of human lymphocytes to low concentrations of radioactive thymidine. *Science*, **223**, 594.

PAINTER, R.B. & YOUNG, B.R. (1980). Radiosensitivity in ataxia telangiectasia: A new explanation. *Proc. Natl Acad. Sci. USA.*, **77**, 7315.

PAINTER, R.B. & YOUNG, B.R. (1987). DNA synthesis in irradiated mammalian cells. *J. Cell Sci.*, Suppl. **6**, 207.

PATERSON, M.C., SMITH, B.P., LOHMAN, P.H.M., ANDERSON, A.K. & FISHMAN, L. (1976). Defective excision repair of $\gamma$-ray damaged DNA in human (ataxia telangiectasia) fibroblasts. *Nature*, **260**, 444.

PHEAR, G., NALBANTOGLU, J. & MEUTH, M. (1987). Next nucleotide effects in mutations driven by DNA precursor pool imbalances at the *aprt* locus of CHO cells. *Proc. Natl Acad. Sci. USA.*, **84**, 4450.

PONNAMPERUMA, C.A., LEMON, R.M., BENNETT, E.L. & CALVIN, M. (1961). Deamination of adenine by ionizing radiation. *Science*, **134**, 113.

PRESTON, R.J. (1982). The use of inhibitors of DNA repair in the study of mechanisms of induction of chromosome aberrations. *Cytogenet. Cell Genet.*, **33**, 20.

PRIVALLE, C.T. & FRIDOVICH, I. (1987). Induction of superoxide dismutase in *Escherichia coli* by heat shock. *Proc. Natl Acad. Sci. USA.*, **84**, 2723.

RAZZAQUE, A., MISZUSANGA, H. & SEIDMAN, M.M. (1983). Rearrangements and mutagenesis of a shuttle vector plasmid after passage in mammalian cells. *Proc. Natl Acad. Sci. USA.*, **80**, 3010.

RESNICK, M.A. & MARTIN, P. (1976). The repair of double-strand breaks in the nuclear DNA of *Saccharomyces cerevisiae* and its genetic control. *Mol. Cell Genet.*, **143**, 119.

ROGINSKI, R.S., SKOULUTCHI, A.I., HENTHORN, P., SMITHIES, O., HSIUNG, N. & KUCHERLAPATI, R. (1983). Coordinate modulation of transfected HSV thymidine kinase and human globin genes. *Cell*, **35**, 149.

ROUET, P. & ESSIGMANN, J.M. (1985). Possible role for thymine glycol in the selective inhibition of DNA synthesis on oxidized DNA templates. *Cancer Res.*, **45**, 6113.

SAIKI, R.K., SCHARF, S., FALOONA, F. & 4 others (1985). Enzymatic amplification of $\beta$-globin genomic sequences and restriction site analysis for diagnosis of sickle cell anaemia. *Science*, **230**, 1350.

SCHAAPER, R.M., KUNKEL, T.A. & LOEB, L. (1983). Infidelity of DNA synthesis is associated with bypass of apurinic sites. *Proc. Natl Acad. Sci. USA.*, **80**, 487.

SCHAPPER, R.M., DANFORTH, B.N. & GLICKMAN, B.W. (1986). Mechanisms of spontaneous mutagenesis: An analysis of the spectrum of spontaneous mutation in the *Escherichia coli* lacI gene. *J. Mol. Biol.*, **189**, 273.

SEEBERG, E. & STEINUM, A.-L. (1980). Repair of X-ray-induced deoxyribonucleic acid single strand breaks in *xth* mutants of *E. coli*. *J. Bacteriol.*, **141**, 1424.

SHADLEY, J.D. & WOLFF, S. (1987). Very low doses of X-rays can cause human lymphocytes to become less susceptible to ionizing radiation. *Mutagenesis*, **2**, 95.

SHIGEMATSU, I. & KAGAN, A. (1986). Cancer in atomic bomb survivors. *GANN Monograph on Cancer Research 32*, Japan Scientific Societies Press, Tokyo and Plenum Press: New York.

SHILOH, Y., COHEN, M.M. & BECKER, Y. (1981). Ataxia telangiectasia: Studies on DNA repair synthesis in fibroblast strains. In *Chromosome Damage and Repair*, Seeberg, E. & Kleppe, K. (eds) p. 361. Plenum Press: New York.

SHILOH, Y., TABOR, E. & BECKER, Y. (1983). Abnormal response of ataxia telangiectasia cells to agents that break the deoxyribose moiety of DNA via a targeted free radical mechanism. *Carcinogenesis*, **4**, 1317.

SIMIC, M.G. & DIZDAROGLU, M. (1985). Formation of radiation-induced cross-links between thymine and tyrosine: Possible model for crosslinking of DNA and proteins by ionizing radiation. *Biochemistry*, **24**, 233.

SKULIMOWSKI, A.W., TURNER, D.R., MORLEY, A.D., SANDERSON, B.J.S. & ITALIANDROS, M. (1986). Molecular basis of X-ray induced mutation at the HPRT locus in human lymphocytes. *Mutat. Res.*, **162**, 105.

SMITH, C.A. (1987). DNA repair in specific sequences in mammalian cells. *J. Cell Sci.*, Suppl. **6**, 225.

SÖDERHALL, S. & LINDAHL, T. (1976). DNA ligases of eukaryotes. *FEBS Lett.*, **67**, 1.

SOGNIER, M.A. & HITTELMAN, W.N. (1983). Loss of repairability of DNA interstrand crosslinks in Fanconi's anaemia with culture age. *Mutat. Res.*, **108**, 383.

STANKOWSKI, L.F. JR. & HSIE, A.W. (1986). Quantitative and molecular analyses of radiation-induced mutation in AS52 cells. *Radiat. Res.*, **105**, 37.

STRAUSS, B., RABKIN, S., SAGHER, D. & MOORE, P. (1982). The role of DNA polymerase in base substitutions mutagenesis on non-instructional templates. *Biochimie*, **64**, 829.

SZOSTAK, J.W., ORR-WEAVER, T.L., ROTHSTEIN, R.J. & STAHL, F.W. (1983). The double-strand-break repair model for recombination. *Cell*, **33**, 25.

TAKESHITA, M., CHANG, C.-N., JOHNSON, F., WILLS, S. & GROLLMAN, A.P. (1987). Oligodeoxynucleotides containing synthetic abasic sites: Model substrates for DNA polymerases and apurinic/apyrimidinic endonucleases. *J. Biol. Chem.*, **262**, 10171.

TAN, K.H., MEYER, D.J., COLES, B. & KETTERER, B. (1986). Thymine hydroperoxide, a substrate for rat Se-dependent glutathion peroxidase and glutathione transferase isoenzymes. *FEBS Lett.*, **207**, 231.

TEEBOR, G.W. & DUKER, N.J. (1975). Human endonuclease activity for DNA apurinic sites. *Nature*, **258**, 544.

TEEBOR, G.W., FRENKEL, K. & GOLDSTEIN, M.S. (1984). Ionizing radiation and tritium transmutation both cause formation of 5-hydroxymethyl-2′-deoxyuridine in cellular DNA. *Proc. Natl Acad. Sci. USA.*, **81**, 318.

TEOULE, R. & CADET, J. (1978). Radiation-induced degradation of the base component in DNA and related substances – final products. In *Effects of Ionizing on DNA*, Hutterman, J. *et al.* (eds) p. 171. Springer Verlag: Berlin.

TEOULE, R., BERT, C. & BONICEL, A. (1977). Thymine fragment damage retained in the DNA polynucleotides chain after gamma irradiation in aerated solution. *Radiat. Res.*, **72**, 190.

THACKER, J. (1985). The molecular nature of mutations in cultured mammalian cells: A review. *Mutat. Res.*, **150**, 431.

THACKER, J. (1986). The use of recombinant DNA techniques to study radiation-induced damage, repair and genetic change in mammalian cells. *Int. J. Radiat. Biol.*, **50**, 1.

THACKER, J. (1986). The nature of mutants induced by ionizing radiation in cultured hamster cells. III. Molecular characterisation of HPRT-deficient mutant induced by $\gamma$-rays or $\alpha$-particles showing that the majority have deletions of all or part of the *hprt* gene. *Mutat. Res.*, **160**, 267.

THACKER, J. & COX, R. (1975). Mutation induced and inactivation in mammalian cells exposed to ionizing radiation. *Nature*, **258**, 429.

THACKER, J. & COX, R. (1983). The relationship between specific chromosome aberrations and radiation-induced mutations in cultured mammalian cells. In *Radiation-Induced Chromosome Damage in Man*, Ishikara, T. & Sasaki, M.S. (eds) p. 235. Alan R. Liss: New York.

THACKER, J., STEPHENS, M.A. & STRETCH, A. (1978). Mutation to ouabain-resistance in Chinese hamster cells: Induction by ethyl methanesulphonate and lack of induction by ionising radiation. *Mutat. Res.*, **51**, 255.

THOMAS, D.C., KUNKEL, T.A., CASNA, N.J., FORD, J.P. & SANCAR, A. (1986). Activities and incision patterns of ABC excinuclease on modified DNA containing single-base mismatches and extra-helical bases. *J. Biol. Chem.*, **261**, 14496.

TINDALL, H.R., STEIN, J. & HUTCHINSON, F. (1988). Changes in DNA base sequence induced by $\gamma$-ray mutagenesis of *lambda* phage and prophage. *Genetics*, in press.

TOTTER, J.R. (1980). Spontaneous cancer and its possible relationship to oxygen metabolism. *Proc. Natl Acad. Sci. USA.*, **77**, 1763.

URLAUB, G., MITCHELL, P.J., KAS, E. & 4 others (1986). Effect of gamma rays at the dihydrofolate reductase locus: Deletions and inversions. *Som. Cell Mol. Genet.*, **12**, 555.

UPTON, A.C. (1984). Biological aspects of radiation carcinogenesis. In *Radiation Carcinogenesis: Epidemiology and Biological Significance*, Boice, J.D. & Fraumeni, J.F. (eds) p. 9. Raven Press: New York.

URLAUB, G., KAS, E., CAROTHERS, A.M. & CHASIN, L.A. (1983). Deletions of the diploid dihydrofolate reductase locus from cultured mammalian cells. *Cell*, **33**, 405.

VAN DER SCHANS, G.P., CENTEN, H.B. & LOHMAN, P.H.M. (1981). Studies on the repair defects of ataxia telangiectasia cells. In *Chromosome Damage and Repair*, Seeberg, E. & Kleppe, K. (eds) p. 355. Plenum Press: New York.

VRIELING, H., SIMONS, J.W.I.M., ARWERT, F., NATARAJAN, A.T. & VAN ZEELAND, A.A. (1985). Mutations induced by X-rays at the HPRT locus in cultured Chinese hamster cells are large deletions. *Mutat. Res.*, **144**, 281.

WAKE, C.T. & WILSON, J.H. (1979). Simian virus 40 recombinants are produced at high frequency during infection with genetically mixed oligomeric DNA. *Proc. Natl Acad. Sci. USA.*, **76**, 2876.

WALKER, G.C. (1984). Mutagenesis and inducible responses to deoxyribonucleic damage in *E. coli*. *Microbiol. Rev.*, **48**, 60.

WARD, J.F. (1985). Biochemistry of DNA lesions. *Radiat. Res.*, **104**, Suppl. 8, S103.

WARD, J.F. & KUO, I. (1976). Strand breaks, base release, and postirradiation changes in DNA $\gamma$-irradiated in dilute $O_2$-saturated aqueous solution. *Radiat. Res.*, **66**, 485.

WEINERT, T.A., DERBYSHIRE, K.M., HUGHSON, F.M. & GRINDLEY, N.D.F. (1984). Replicative and conservative transpositional recombination of insertion sequences. *Cold Spring Harbor Symp. Quant. Biol.*, **49**, 251.

WILLIS, A.E. & LINDAHL, T. (1987). DNA ligase I deficiency in Bloom's syndrome. *Nature*, **325**, 355.

WILSON, J.M., STOUT, J.T., PALELLA, T.D., DAVIDSON, B.L., KELLEY, N.N. & CASKEY, C.T. (1986). A molecular survey of hypoxanthine-guanine phospho-ribosyltransferase deficiency in man. *J. Clin. Invest.*, **77**, 188.

WITKIN, E.M. (1976). Ultraviolet mutagenesis and inducible DNA repair in *E. coli. Bacteriol. Rev.*, **40**, 869.

WOOD, R.D., BURKI, H.J., HUGHES, M. & POLEY, A. (1983). Radiation induced lethality and mutation in a repair-deficient CHO cell line. *Int. J. Radiat. Biol.*, **43**, 207.

WOOD, W.I., GUTSCHIER, J., LASKY, L.A. & LAWN, R.M. (1985). Base composition independent hybridization in tetramethyl-ammonium chloride: A method for oligonucleotide screening of highly complex gene libraries. *Proc. Natl Acad. Sci. USA.*, **82**, 1585.

YANDELL, D.W., DRYA, T.P. & LITTLE, J.B. (1986). Somatic mutations at a heterozygous autosomal locus in human cells occur more frequently by allele loss than by intragenic structural alterations. *Som. Cell Mol. Genet.*, **12**, 255.

YANDELL, D.W., DRYA, T.P. & LITTLE, J.B. (1987). Molecular genetic analysis of recessive mutations at a heterozygous autosomal locus in human cells. *Mol. Cell Biol.*, (in press).

YANG, T.P., PATEL, P.I., CHINAULT, A.C. & 4 others (1984). Molecular evidence for new mutations at the HPRT locus in Lesch–Nyan patients. *Nature*, **310**, 412.

YATAGAI, F., HORSFALL, M.J. & GLICKMAN, B.W. (1987). Defect in excision repair alters the mutational specificity of PUVA treatment in the *lac I* gene of *E. coli. J. Mol. Biol.*, **194**, 601.


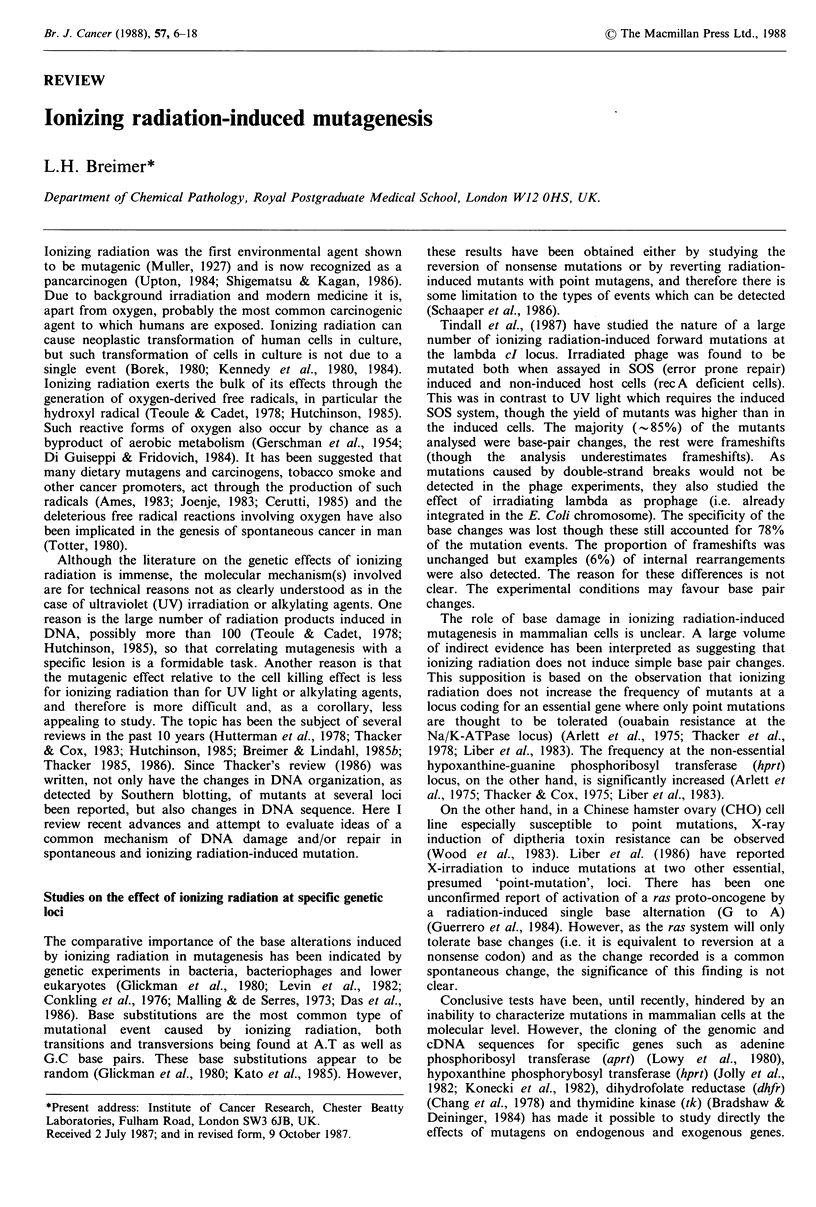

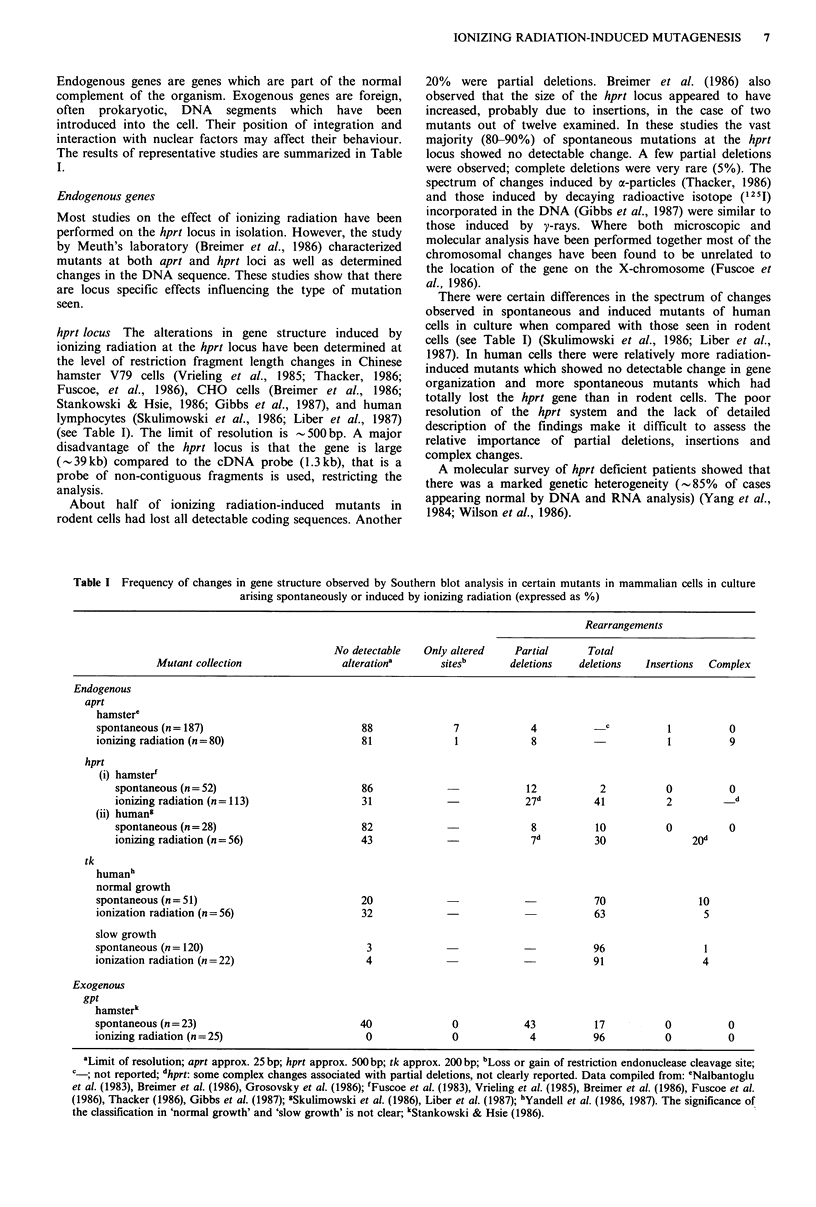

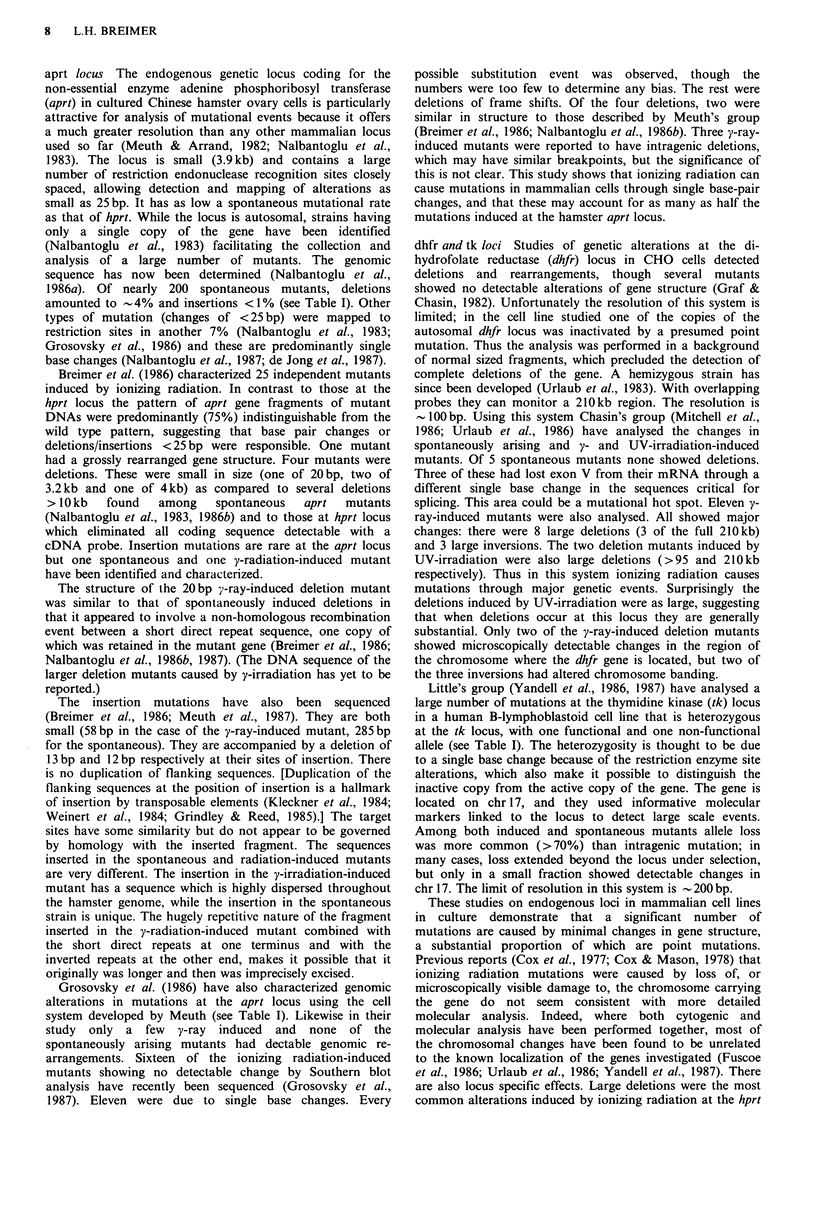

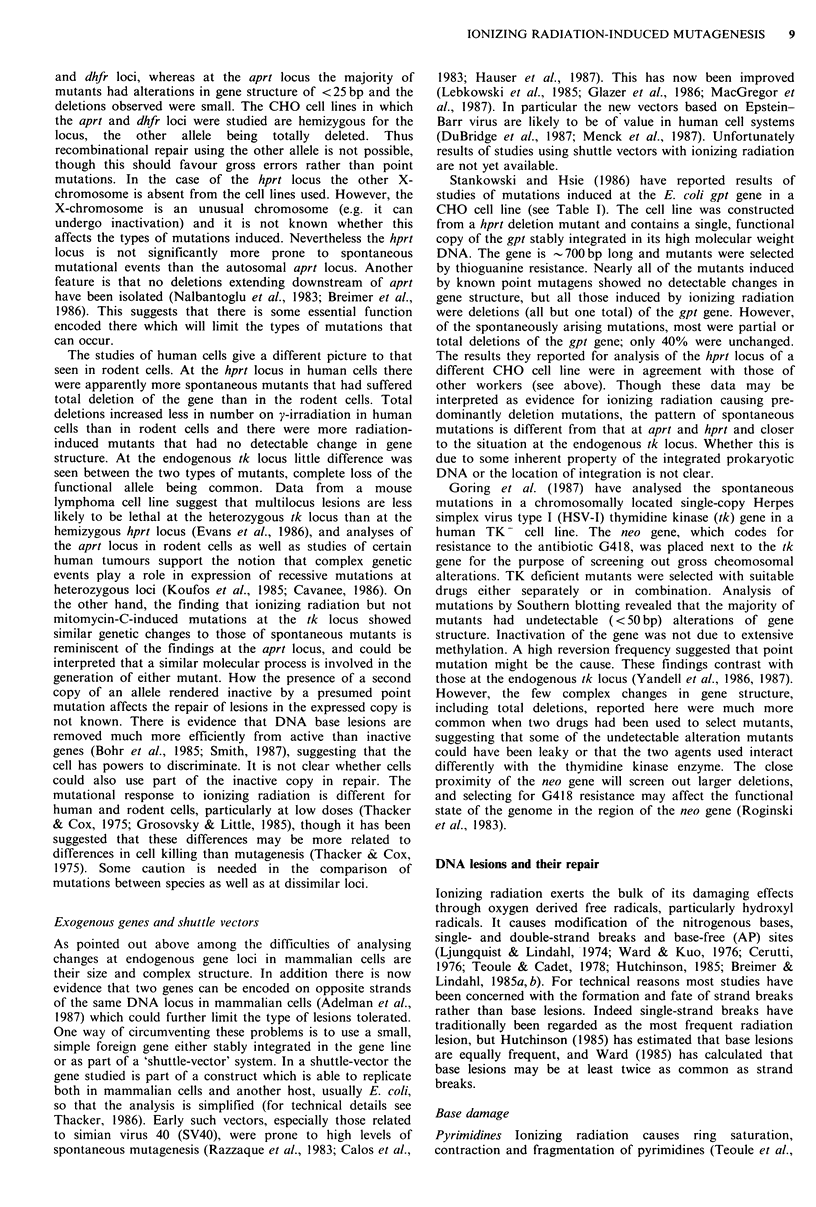

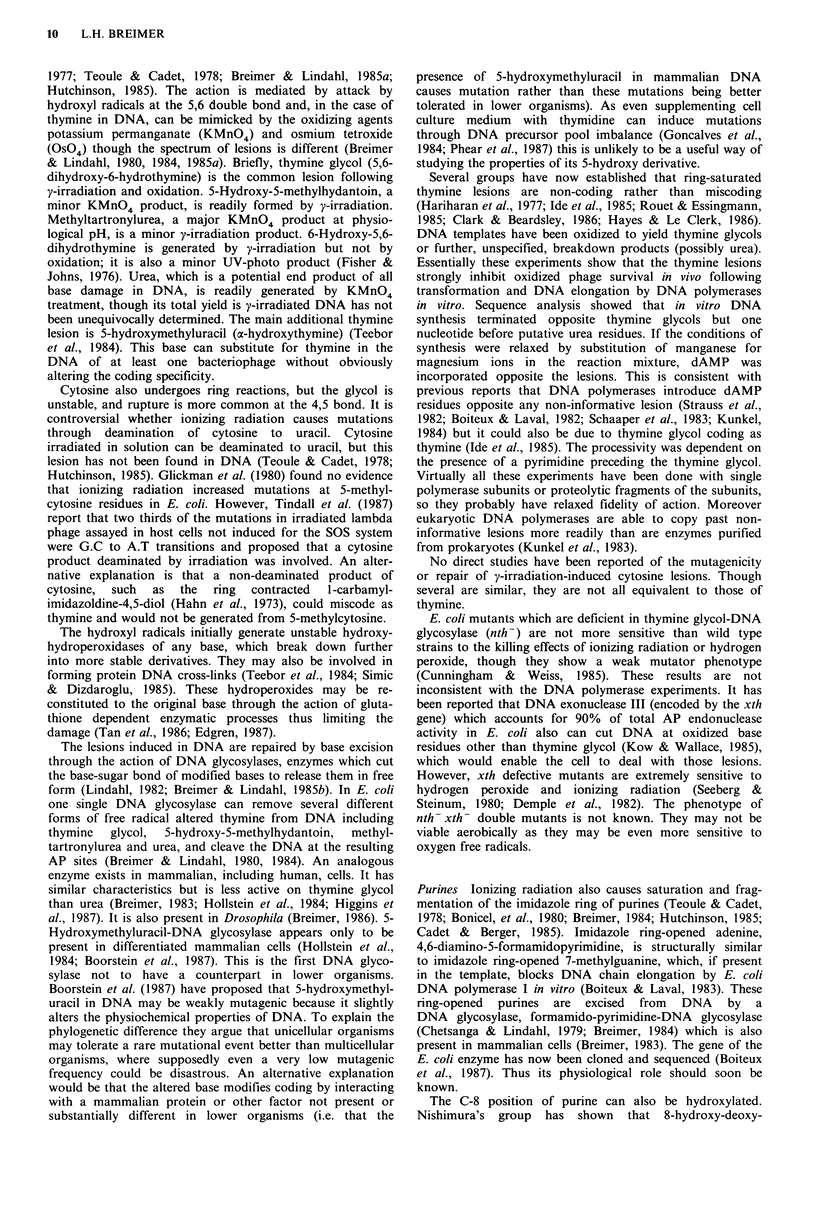

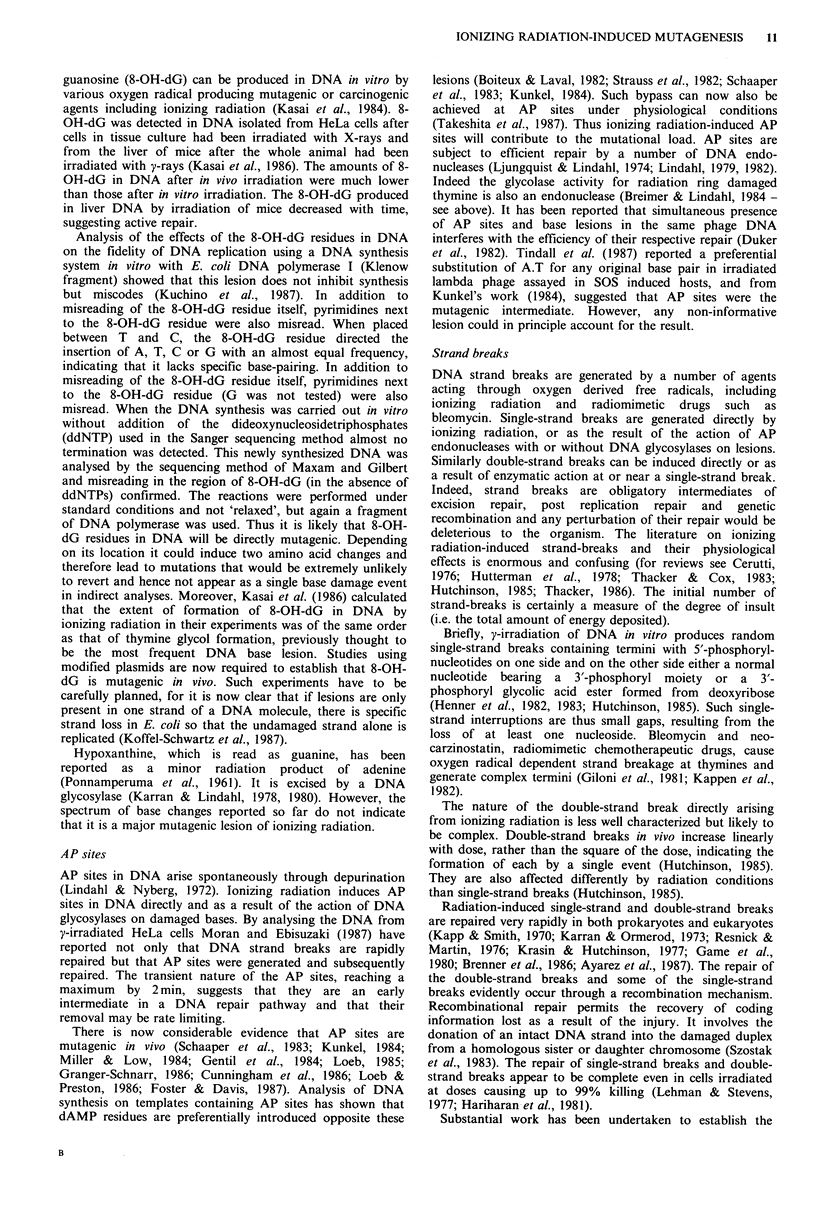

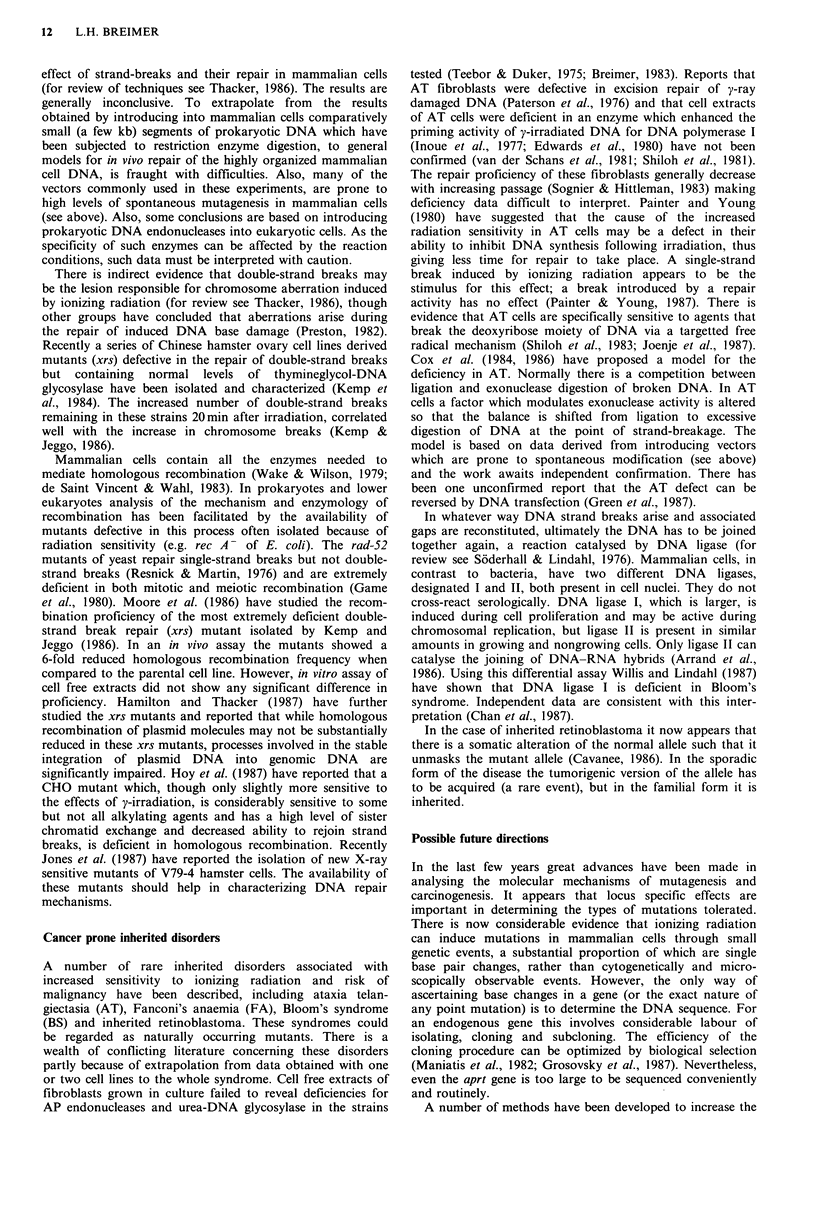

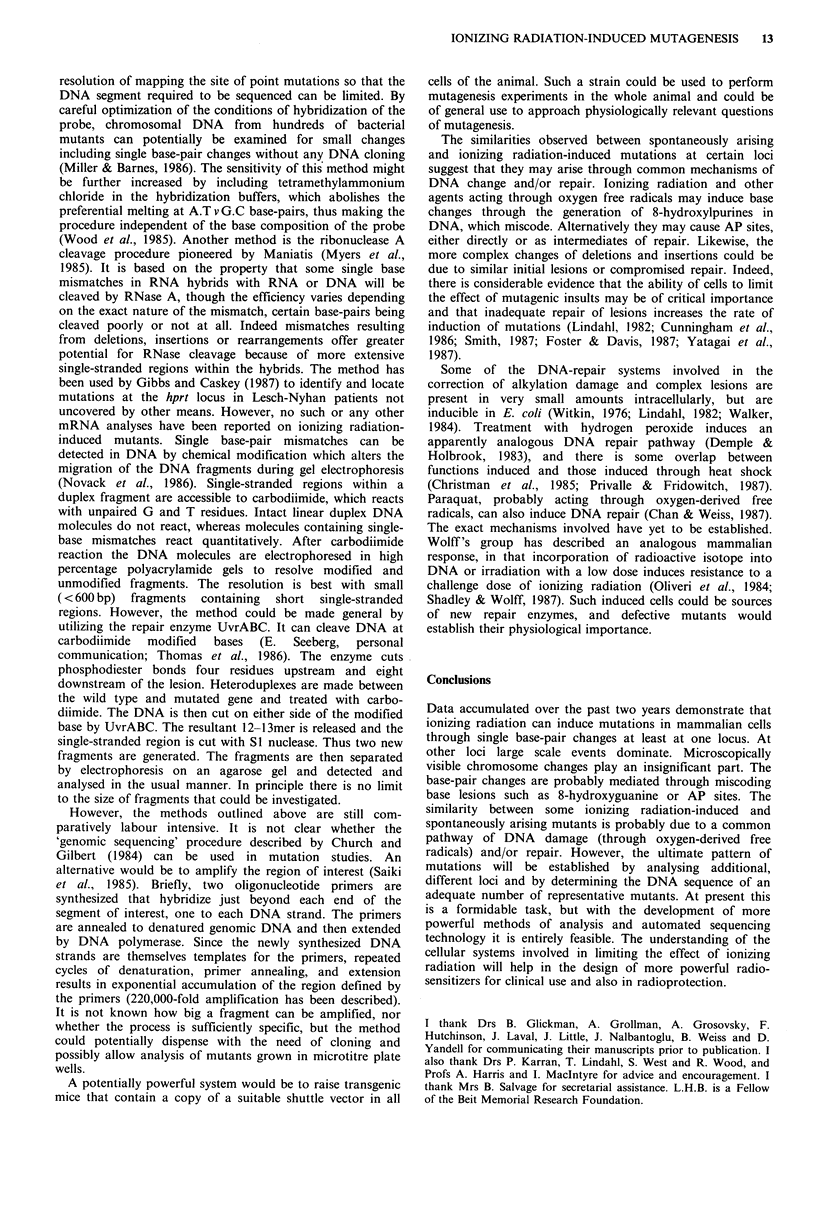

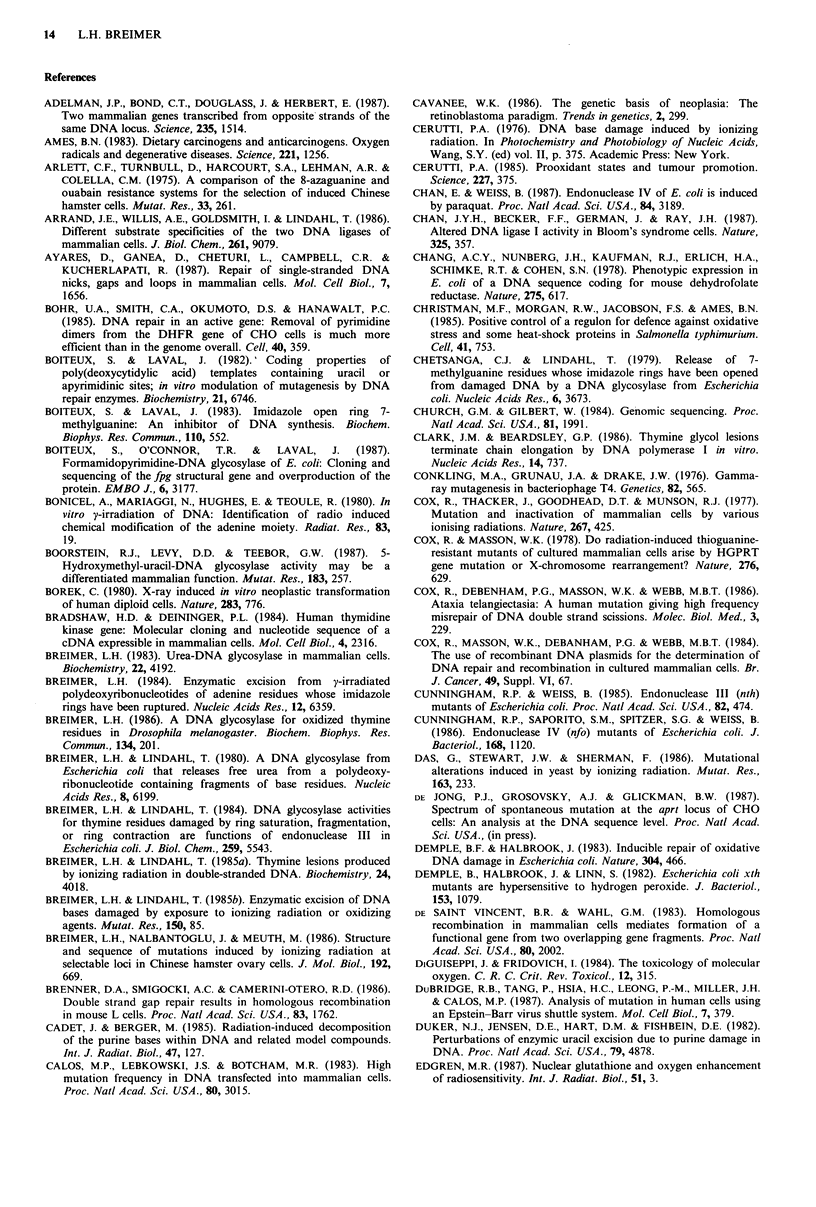

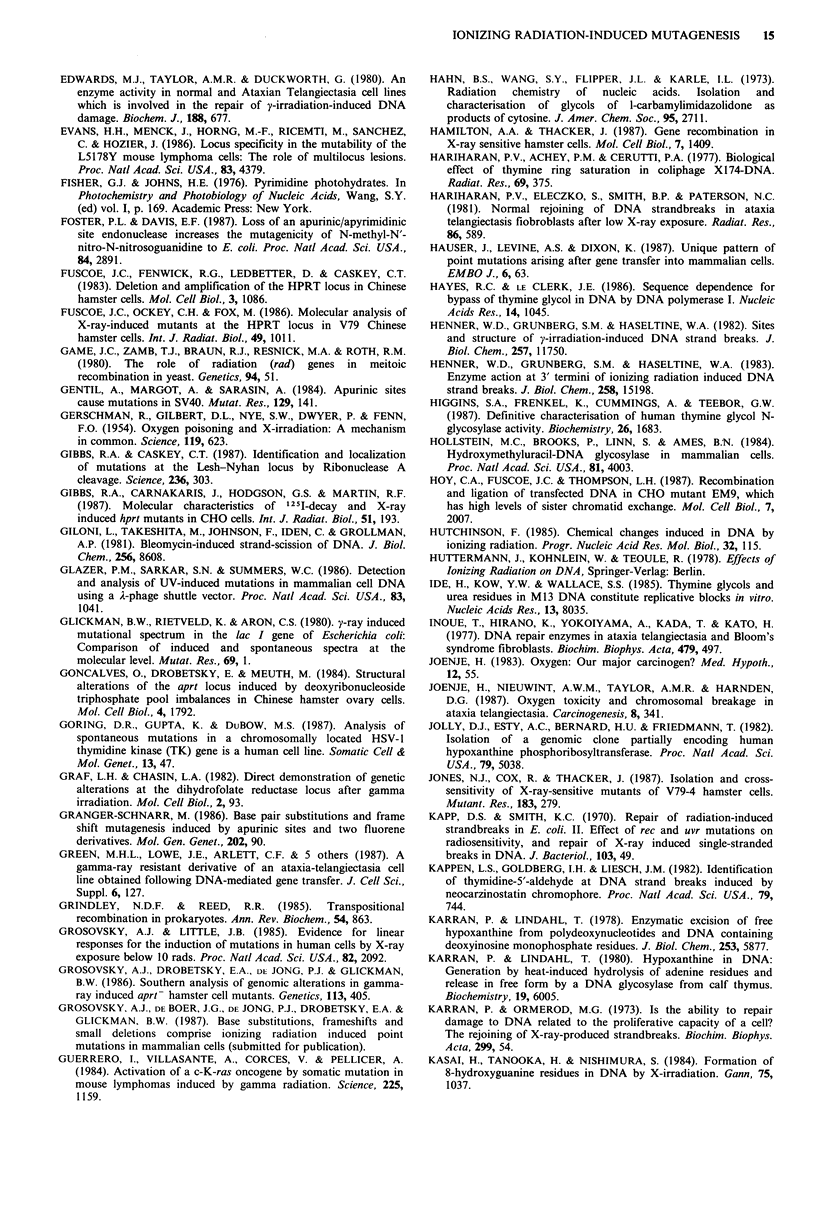

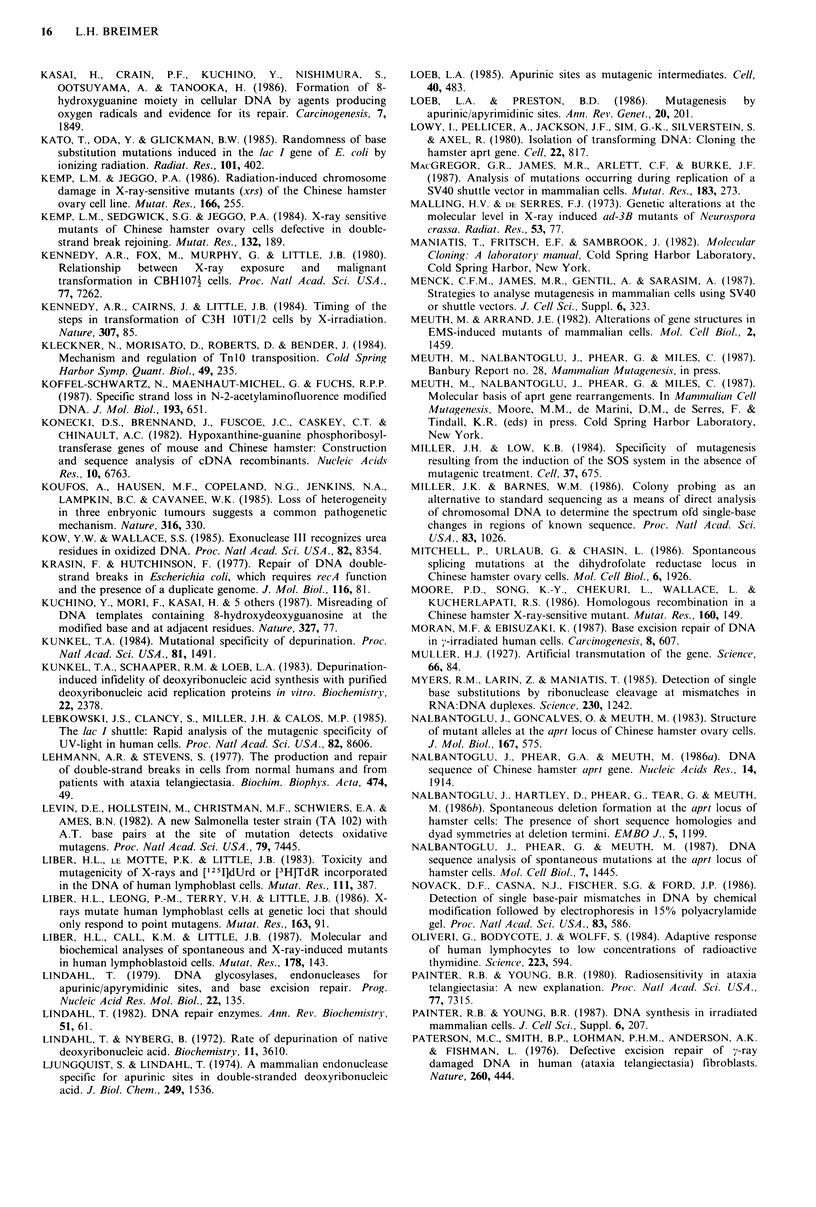

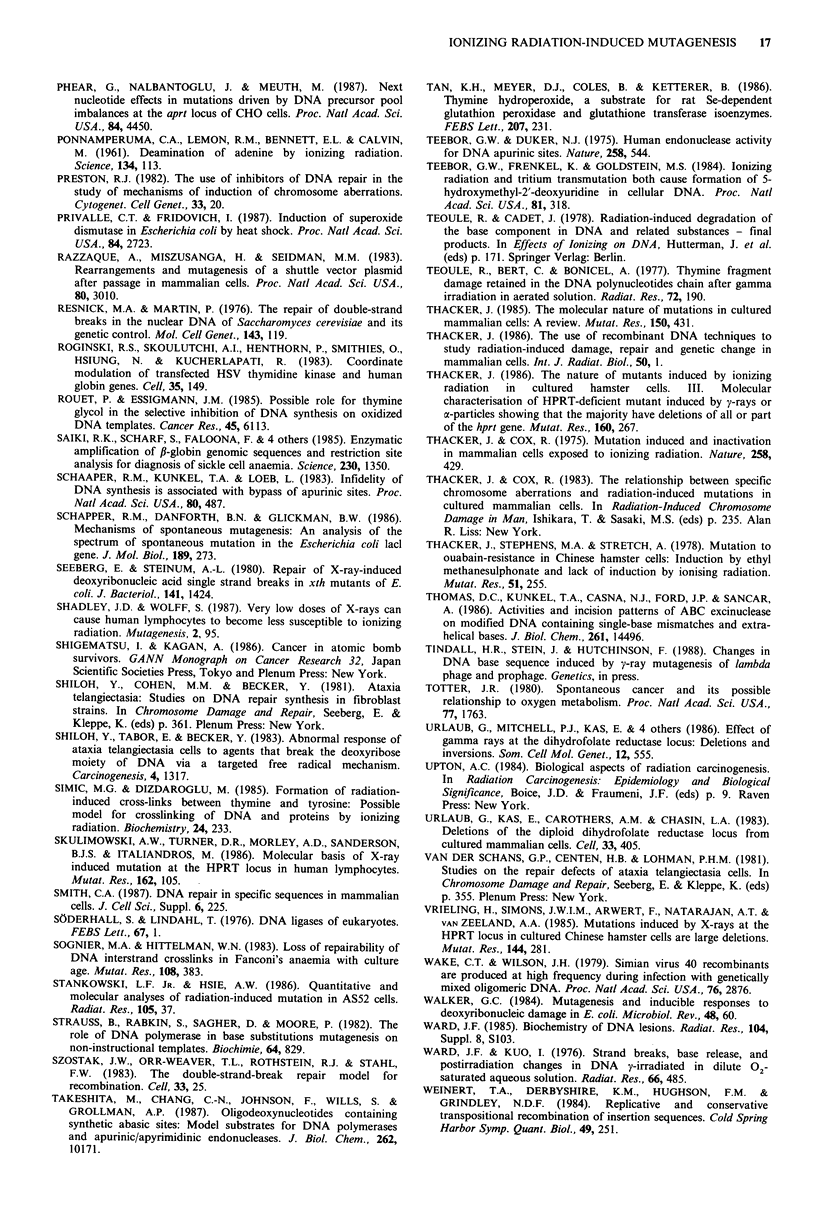

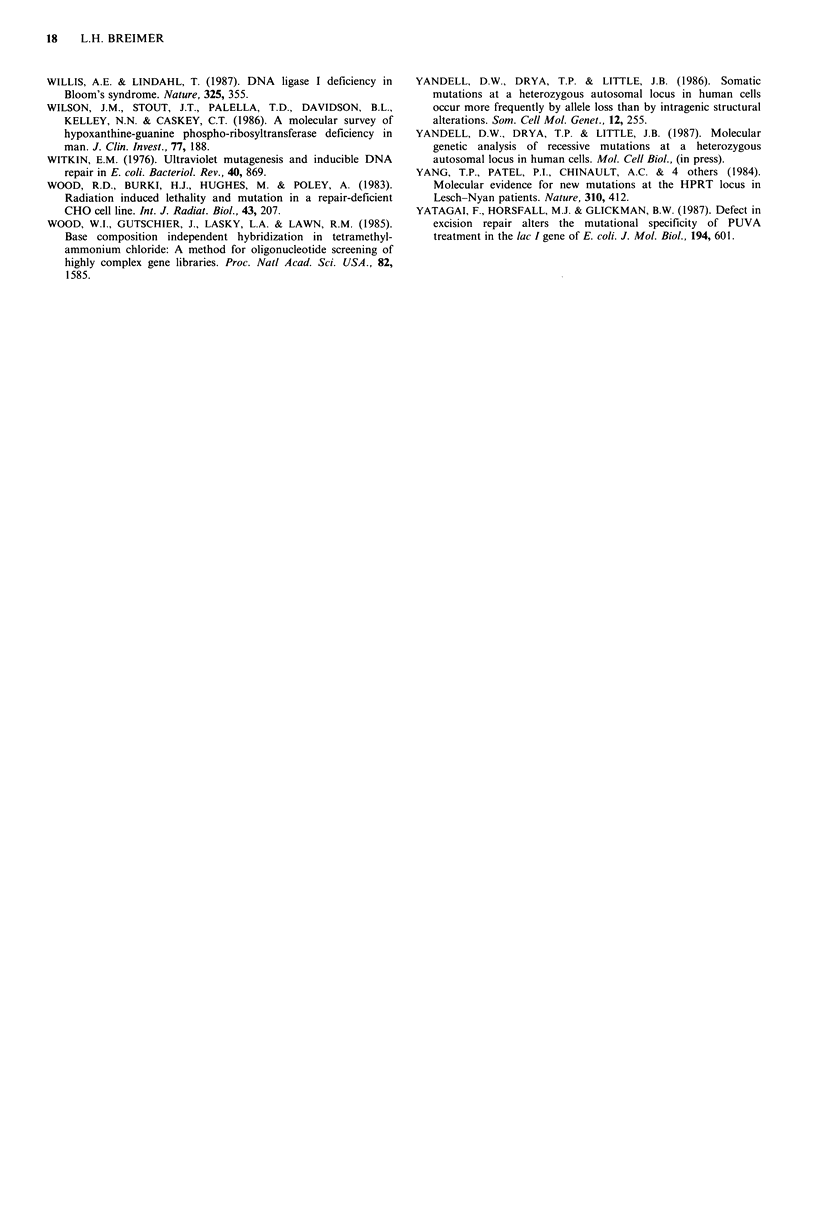


## References

[OCR_01220] Adelman J. P., Bond C. T., Douglass J., Herbert E. (1987). Two mammalian genes transcribed from opposite strands of the same DNA locus.. Science.

[OCR_01225] Ames B. N. (1983). Dietary carcinogens and anticarcinogens. Oxygen radicals and degenerative diseases.. Science.

[OCR_01229] Arlett C. F., Turnbull D., Harcourt S. A., Lehmann A. R., Colella C. M. (1975). A comparison of the 8-azaguanine and ouabain-resistance systems for the selection of induced mutant Chinese hamster cells.. Mutat Res.

[OCR_01235] Arrand J. E., Willis A. E., Goldsmith I., Lindahl T. (1986). Different substrate specificities of the two DNA ligases of mammalian cells.. J Biol Chem.

[OCR_01240] Ayares D., Ganea D., Chekuri L., Campbell C. R., Kucherlapati R. (1987). Repair of single-stranded DNA nicks, gaps, and loops in mammalian cells.. Mol Cell Biol.

[OCR_01246] Bohr V. A., Smith C. A., Okumoto D. S., Hanawalt P. C. (1985). DNA repair in an active gene: removal of pyrimidine dimers from the DHFR gene of CHO cells is much more efficient than in the genome overall.. Cell.

[OCR_01252] Boiteux S., Laval J. (1982). Coding properties of poly(deoxycytidylic acid) templates containing uracil or apyrimidinic sites: in vitro modulation of mutagenesis by deoxyribonucleic acid repair enzymes.. Biochemistry.

[OCR_01258] Boiteux S., Laval J. (1983). Imidazole open ring 7-methylguanine: an inhibitor of DNA synthesis.. Biochem Biophys Res Commun.

[OCR_01263] Boiteux S., O'Connor T. R., Laval J. (1987). Formamidopyrimidine-DNA glycosylase of Escherichia coli: cloning and sequencing of the fpg structural gene and overproduction of the protein.. EMBO J.

[OCR_01269] Bonicel A., Mariaggi N., Hughes E., Teoule R. (1980). In vitro gamma irradiation of DNA: identification of radioinduced chemical modifications of the adenine moiety.. Radiat Res.

[OCR_01275] Boorstein R. J., Levy D. D., Teebor G. W. (1987). 5-Hydroxymethyluracil-DNA glycosylase activity may be a differentiated mammalian function.. Mutat Res.

[OCR_01280] Borek C. (1980). X-ray induced in vitro neoplastic transformation of human diploid cells.. Nature.

[OCR_01284] Bradshaw H. D., Deininger P. L. (1984). Human thymidine kinase gene: molecular cloning and nucleotide sequence of a cDNA expressible in mammalian cells.. Mol Cell Biol.

[OCR_01298] Breimer L. H. (1986). A DNA glycosylase for oxidized thymine residues in Drosophila melanogaster.. Biochem Biophys Res Commun.

[OCR_01293] Breimer L. H. (1984). Enzymatic excision from gamma-irradiated polydeoxyribonucleotides of adenine residues whose imidazole rings have been ruptured.. Nucleic Acids Res.

[OCR_01309] Breimer L. H., Lindahl T. (1984). DNA glycosylase activities for thymine residues damaged by ring saturation, fragmentation, or ring contraction are functions of endonuclease III in Escherichia coli.. J Biol Chem.

[OCR_01320] Breimer L. H., Lindahl T. (1985). Enzymatic excision of DNA bases damaged by exposure to ionizing radiation or oxidizing agents.. Mutat Res.

[OCR_01315] Breimer L. H., Lindahl T. (1985). Thymine lesions produced by ionizing radiation in double-stranded DNA.. Biochemistry.

[OCR_01325] Breimer L. H., Nalbantoglu J., Meuth M. (1986). Structure and sequence of mutations induced by ionizing radiation at selectable loci in Chinese hamster ovary cells.. J Mol Biol.

[OCR_01289] Breimer L. H. (1983). Urea--DNA glycosylase in mammalian cells.. Biochemistry.

[OCR_01303] Breimer L., Lindahl T. (1980). A DNA glycosylase from Escherichia coli that releases free urea from a polydeoxyribonucleotide containing fragments of base residues.. Nucleic Acids Res.

[OCR_01331] Brenner D. A., Smigocki A. C., Camerini-Otero R. D. (1986). Double-strand gap repair results in homologous recombination in mouse L cells.. Proc Natl Acad Sci U S A.

[OCR_01336] Cadet J., Berger M. (1985). Radiation-induced decomposition of the purine bases within DNA and related model compounds.. Int J Radiat Biol Relat Stud Phys Chem Med.

[OCR_01341] Calos M. P., Lebkowski J. S., Botchan M. R. (1983). High mutation frequency in DNA transfected into mammalian cells.. Proc Natl Acad Sci U S A.

[OCR_01355] Cerutti P. A. (1985). Prooxidant states and tumor promotion.. Science.

[OCR_01359] Chan E., Weiss B. (1987). Endonuclease IV of Escherichia coli is induced by paraquat.. Proc Natl Acad Sci U S A.

[OCR_01363] Chan J. Y., Becker F. F., German J., Ray J. H. (1987). Altered DNA ligase I activity in Bloom's syndrome cells.. Nature.

[OCR_01368] Chang A. C., Nunberg J. H., Kaufman R. J., Erlich H. A., Schimke R. T., Cohen S. N. (1978). Phenotypic expression in E. coli of a DNA sequence coding for mouse dihydrofolate reductase.. Nature.

[OCR_01380] Chetsanga C. J., Lindahl T. (1979). Release of 7-methylguanine residues whose imidazole rings have been opened from damaged DNA by a DNA glycosylase from Escherichia coli.. Nucleic Acids Res.

[OCR_01374] Christman M. F., Morgan R. W., Jacobson F. S., Ames B. N. (1985). Positive control of a regulon for defenses against oxidative stress and some heat-shock proteins in Salmonella typhimurium.. Cell.

[OCR_01386] Church G. M., Gilbert W. (1984). Genomic sequencing.. Proc Natl Acad Sci U S A.

[OCR_01390] Clark J. M., Beardsley G. P. (1986). Thymine glycol lesions terminate chain elongation by DNA polymerase I in vitro.. Nucleic Acids Res.

[OCR_01395] Conkling M. A., Grunau J. A., Drake J. W. (1976). Gamma-ray mutagenesis in bacteriophage T4.. Genetics.

[OCR_01410] Cox R., Debenham P. G., Masson W. K., Webb M. B. (1986). Ataxia-telangiectasia: a human mutation giving high-frequency misrepair of DNA double-stranded scissions.. Mol Biol Med.

[OCR_01404] Cox R., Masson W. K. (1978). Do radiation-induced thioguanine-resistant mutants of cultured mammalian cells arise by HGPRT gene mutation or X-chromosome rearrangement?. Nature.

[OCR_01399] Cox R., Thacker J., Goodhead D. T., Munson R. J. (1977). Mutation and inactivation of mammalian cells by various ionising radiations.. Nature.

[OCR_01426] Cunningham R. P., Saporito S. M., Spitzer S. G., Weiss B. (1986). Endonuclease IV (nfo) mutant of Escherichia coli.. J Bacteriol.

[OCR_01422] Cunningham R. P., Weiss B. (1985). Endonuclease III (nth) mutants of Escherichia coli.. Proc Natl Acad Sci U S A.

[OCR_01431] Das G., Stewart J. W., Sherman F. (1986). Mutational alterations induced in yeast by ionizing radiation.. Mutat Res.

[OCR_01442] Demple B., Halbrook J. (1983). Inducible repair of oxidative DNA damage in Escherichia coli.. Nature.

[OCR_01446] Demple B., Halbrook J., Linn S. (1983). Escherichia coli xth mutants are hypersensitive to hydrogen peroxide.. J Bacteriol.

[OCR_01457] DiGuiseppi J., Fridovich I. (1984). The toxicology of molecular oxygen.. Crit Rev Toxicol.

[OCR_01461] DuBridge R. B., Tang P., Hsia H. C., Leong P. M., Miller J. H., Calos M. P. (1987). Analysis of mutation in human cells by using an Epstein-Barr virus shuttle system.. Mol Cell Biol.

[OCR_01466] Duker N. J., Jensen D. E., Hart D. M., Fishbein D. E. (1982). Perturbations of enzymic uracil excision due to purine damage in DNA.. Proc Natl Acad Sci U S A.

[OCR_01471] Edgren M. R. (1987). Nuclear glutathione and oxygen enhancement of radiosensitivity.. Int J Radiat Biol Relat Stud Phys Chem Med.

[OCR_01477] Edwards M. J., Taylor A. M., Duckworth G. (1980). An enzyme activity in normal and ataxia telangiectasia cell lines which is involved in the repair of gamma-irradiation-induced DNA damage.. Biochem J.

[OCR_01483] Evans H. H., Mencl J., Horng M. F., Ricanati M., Sanchez C., Hozier J. (1986). Locus specificity in the mutability of L5178Y mouse lymphoma cells: the role of multilocus lesions.. Proc Natl Acad Sci U S A.

[OCR_01494] Foster P. L., Davis E. F. (1987). Loss of an apurinic/apyrimidinic site endonuclease increases the mutagenicity of N-methyl-N'-nitro-N-nitrosoguanidine to Escherichia coli.. Proc Natl Acad Sci U S A.

[OCR_01500] Fuscoe J. C., Fenwick R. G., Ledbetter D. H., Caskey C. T. (1983). Deletion and amplification of the HGPRT locus in Chinese hamster cells.. Mol Cell Biol.

[OCR_01505] Fuscoe J. C., Ockey C. H., Fox M. (1986). Molecular analysis of X-ray-induced mutants at the HPRT locus in V79 Chinese hamster cells.. Int J Radiat Biol Relat Stud Phys Chem Med.

[OCR_01519] GERSCHMAN R., GILBERT D. L., NYE S. W., DWYER P., FENN W. O. (1954). Oxygen poisoning and x-irradiation: a mechanism in common.. Science.

[OCR_01510] Game J. C., Zamb T. J., Braun R. J., Resnick M., Roth R. M. (1980). The Role of Radiation (rad) Genes in Meiotic Recombination in Yeast.. Genetics.

[OCR_01515] Gentil A., Margot A., Sarasin A. (1984). Apurinic sites cause mutations in simian virus 40.. Mutat Res.

[OCR_01529] Gibbs R. A., Camakaris J., Hodgson G. S., Martin R. F. (1987). Molecular characterization of 125I decay and X-ray-induced HPRT mutants in CHO cells.. Int J Radiat Biol Relat Stud Phys Chem Med.

[OCR_01524] Gibbs R. A., Caskey C. T. (1987). Identification and localization of mutations at the Lesch-Nyhan locus by ribonuclease A cleavage.. Science.

[OCR_01534] Giloni L., Takeshita M., Johnson F., Iden C., Grollman A. P. (1981). Bleomycin-induced strand-scission of DNA. Mechanism of deoxyribose cleavage.. J Biol Chem.

[OCR_01539] Glazer P. M., Sarkar S. N., Summers W. C. (1986). Detection and analysis of UV-induced mutations in mammalian cell DNA using a lambda phage shuttle vector.. Proc Natl Acad Sci U S A.

[OCR_01551] Goncalves O., Drobetsky E., Meuth M. (1984). Structural alterations of the aprt locus induced by deoxyribonucleoside triphosphate pool imbalances in Chinese hamster ovary cells.. Mol Cell Biol.

[OCR_01557] Goring D. R., Gupta K., DuBow M. S. (1987). Analysis of spontaneous mutations in a chromosomally located HSV-1 thymidine kinase (TK) gene in a human cell line.. Somat Cell Mol Genet.

[OCR_01563] Graf L. H., Chasin L. A. (1982). Direct demonstration of genetic alterations at the dihydrofolate reductase locus after gamma irradiation.. Mol Cell Biol.

[OCR_01568] Granger-Schnarr M. (1986). Base pair substitution and frameshift mutagenesis induced by apurinic sites and two fluorene derivatives in a recA441 lexA (Def) strain.. Mol Gen Genet.

[OCR_01573] Green M. H., Lowe J. E., Arlett C. F., Harcourt S. A., Burke J. F., James M. R., Lehmann A. R., Povey S. M. (1987). A gamma-ray-resistant derivative of an ataxia telangiectasia cell line obtained following DNA-mediated gene transfer.. J Cell Sci Suppl.

[OCR_01579] Grindley N. D., Reed R. R. (1985). Transpositional recombination in prokaryotes.. Annu Rev Biochem.

[OCR_01588] Grosovsky A. J., Drobetsky E. A., deJong P. J., Glickman B. W. (1986). Southern analysis of genomic alterations in gamma-ray-induced aprt- hamster cell mutants.. Genetics.

[OCR_01583] Grosovsky A. J., Little J. B. (1985). Evidence for linear response for the induction of mutations in human cells by x-ray exposures below 10 rads.. Proc Natl Acad Sci U S A.

[OCR_01599] Guerrero I., Villasante A., Corces V., Pellicer A. (1984). Activation of a c-K-ras oncogene by somatic mutation in mouse lymphomas induced by gamma radiation.. Science.

[OCR_01605] Hahn B. S., Wang S. Y., Flippen J. L., Karle I. L. (1973). Radiation chemistry of nucleic acids. Isolation and characterization of glycols of 1-carbamylimidazolidone as products of cytosine.. J Am Chem Soc.

[OCR_01611] Hamilton A. A., Thacker J. (1987). Gene recombination in X-ray-sensitive hamster cells.. Mol Cell Biol.

[OCR_01615] Hariharan P. V., Achey P. M., Cerutti P. A. (1977). Biological effect of thymine ring saturation in coliphage phiX174-DNA.. Radiat Res.

[OCR_01620] Hariharan P. V., Eleczko S., Smith B. P., Paterson M. C. (1981). Normal rejoining of DNA strand breaks in ataxia telangiectasia fibroblast lines after low x-ray exposure.. Radiat Res.

[OCR_01626] Hauser J., Levine A. S., Dixon K. (1987). Unique pattern of point mutations arising after gene transfer into mammalian cells.. EMBO J.

[OCR_01631] Hayes R. C., LeClerc J. E. (1986). Sequence dependence for bypass of thymine glycols in DNA by DNA polymerase I.. Nucleic Acids Res.

[OCR_01641] Henner W. D., Grunberg S. M., Haseltine W. A. (1983). Enzyme action at 3' termini of ionizing radiation-induced DNA strand breaks.. J Biol Chem.

[OCR_01636] Henner W. D., Grunberg S. M., Haseltine W. A. (1982). Sites and structure of gamma radiation-induced DNA strand breaks.. J Biol Chem.

[OCR_01646] Higgins S. A., Frenkel K., Cummings A., Teebor G. W. (1987). Definitive characterization of human thymine glycol N-glycosylase activity.. Biochemistry.

[OCR_01651] Hollstein M. C., Brooks P., Linn S., Ames B. N. (1984). Hydroxymethyluracil DNA glycosylase in mammalian cells.. Proc Natl Acad Sci U S A.

[OCR_01656] Hoy C. A., Fuscoe J. C., Thompson L. H. (1987). Recombination and ligation of transfected DNA in CHO mutant EM9, which has high levels of sister chromatid exchange.. Mol Cell Biol.

[OCR_01662] Hutchinson F. (1985). Chemical changes induced in DNA by ionizing radiation.. Prog Nucleic Acid Res Mol Biol.

[OCR_01670] Ide H., Kow Y. W., Wallace S. S. (1985). Thymine glycols and urea residues in M13 DNA constitute replicative blocks in vitro.. Nucleic Acids Res.

[OCR_01675] Inoue T., Hirano K., Yokoiyama A., Kada T., Kato H. (1977). DNA repair enzymes in ataxia telangiectasia and Bloom's syndrome fibroblasts.. Biochim Biophys Acta.

[OCR_01684] Joenje H., Nieuwint A. W., Taylor A. M., Harnden D. G. (1987). Oxygen toxicity and chromosomal breakage in ataxia telangiectasia.. Carcinogenesis.

[OCR_01680] Joenje H. (1983). Oxygen: our major carcinogen?. Med Hypotheses.

[OCR_01689] Jolly D. J., Esty A. C., Bernard H. U., Friedmann T. (1982). Isolation of a genomic clone partially encoding human hypoxanthine phosphoribosyltransferase.. Proc Natl Acad Sci U S A.

[OCR_01695] Jones N. J., Cox R., Thacker J. (1987). Isolation and cross-sensitivity of X-ray-sensitive mutants of V79-4 hamster cells.. Mutat Res.

[OCR_01700] Kapp D. S., Smith K. C. (1970). Repair of radiation-induced damage in Escherichia coli. II. Effect of rec and uvr mutations on radiosensitivity, and repair of x-ray-induced single-strand breaks in deoxyribonucleic acid.. J Bacteriol.

[OCR_01706] Kappen L. S., Goldberg I. H., Liesch J. M. (1982). Identification of thymidine-5'-aldehyde at DNA strand breaks induced by neocarzinostatin chromophore.. Proc Natl Acad Sci U S A.

[OCR_01712] Karran P., Lindahl T. (1978). Enzymatic excision of free hypoxanthine from polydeoxynucleotides and DNA containing deoxyinosine monophosphate residues.. J Biol Chem.

[OCR_01717] Karran P., Lindahl T. (1980). Hypoxanthine in deoxyribonucleic acid: generation by heat-induced hydrolysis of adenine residues and release in free form by a deoxyribonucleic acid glycosylase from calf thymus.. Biochemistry.

[OCR_01723] Karran P., Ormerod M. G. (1973). Is the ability to repair damage to DNA related to the proliferative capacity of a cell? The rejoining of X-ray-produced strand breaks.. Biochim Biophys Acta.

[OCR_01736] Kasai H., Crain P. F., Kuchino Y., Nishimura S., Ootsuyama A., Tanooka H. (1986). Formation of 8-hydroxyguanine moiety in cellular DNA by agents producing oxygen radicals and evidence for its repair.. Carcinogenesis.

[OCR_01729] Kasai H., Tanooka H., Nishimura S. (1984). Formation of 8-hydroxyguanine residues in DNA by X-irradiation.. Gan.

[OCR_01743] Kato T., Oda Y., Glickman B. W. (1985). Randomness of base substitution mutations induced in the lacI gene of Escherichia coli by ionizing radiation.. Radiat Res.

[OCR_01748] Kemp L. M., Jeggo P. A. (1986). Radiation-induced chromosome damage in X-ray-sensitive mutants (xrs) of the Chinese hamster ovary cell line.. Mutat Res.

[OCR_01753] Kemp L. M., Sedgwick S. G., Jeggo P. A. (1984). X-ray sensitive mutants of Chinese hamster ovary cells defective in double-strand break rejoining.. Mutat Res.

[OCR_01764] Kennedy A. R., Cairns J., Little J. B. (1984). Timing of the steps in transformation of C3H 10T 1/2 cells by X-irradiation.. Nature.

[OCR_01758] Kennedy A. R., Fox M., Murphy G., Little J. B. (1980). Relationship between x-ray exposure and malignant transformation in C3H 10T1/2 cells.. Proc Natl Acad Sci U S A.

[OCR_01769] Kleckner N., Morisato D., Roberts D., Bender J. (1984). Mechanism and regulation of Tn10 transposition.. Cold Spring Harb Symp Quant Biol.

[OCR_01774] Koffel-Schwartz N., Maenhaut-Michel G., Fuchs R. P. (1987). Specific strand loss in N-2-acetylaminofluorene-modified DNA.. J Mol Biol.

[OCR_01779] Konecki D. S., Brennand J., Fuscoe J. C., Caskey C. T., Chinault A. C. (1982). Hypoxanthine-guanine phosphoribosyltransferase genes of mouse and Chinese hamster: construction and sequence analysis of cDNA recombinants.. Nucleic Acids Res.

[OCR_01786] Koufos A., Hansen M. F., Copeland N. G., Jenkins N. A., Lampkin B. C., Cavenee W. K. (1985). Loss of heterozygosity in three embryonal tumours suggests a common pathogenetic mechanism.. Nature.

[OCR_01792] Kow Y. W., Wallace S. S. (1985). Exonuclease III recognizes urea residues in oxidized DNA.. Proc Natl Acad Sci U S A.

[OCR_01796] Krasin F., Hutchinson F. (1977). Repair of DNA double-strand breaks in Escherichia coli, which requires recA function and the presence of a duplicate genome.. J Mol Biol.

[OCR_01810] Kunkel T. A., Schaaper R. M., Loeb L. A. (1983). Depurination-induced infidelity of deoxyribonucleic acid synthesis with purified deoxyribonucleic acid replication proteins in vitro.. Biochemistry.

[OCR_01816] Lebkowski J. S., Clancy S., Miller J. H., Calos M. P. (1985). The lacI shuttle: rapid analysis of the mutagenic specificity of ultraviolet light in human cells.. Proc Natl Acad Sci U S A.

[OCR_01821] Lehman A. R., Stevens S. (1977). The production and repair of double strand breaks in cells from normal humans and from patients with ataxia telangiectasia.. Biochim Biophys Acta.

[OCR_01827] Levin D. E., Hollstein M., Christman M. F., Schwiers E. A., Ames B. N. (1982). A new Salmonella tester strain (TA102) with A X T base pairs at the site of mutation detects oxidative mutagens.. Proc Natl Acad Sci U S A.

[OCR_01843] Liber H. L., Call K. M., Little J. B. (1987). Molecular and biochemical analyses of spontaneous and X-ray-induced mutants in human lymphoblastoid cells.. Mutat Res.

[OCR_01833] Liber H. L., LeMotte P. K., Little J. B. (1983). Toxicity and mutagenicity of X-rays and [125I]dUrd or [3H]TdR incorporated in the DNA of human lymphoblast cells.. Mutat Res.

[OCR_01838] Liber H. L., Leong P. M., Terry V. H., Little J. B. (1986). X-rays mutate human lymphoblast cells at genetic loci that should respond only to point mutagens.. Mutat Res.

[OCR_01848] Lindahl T. (1979). DNA glycosylases, endonucleases for apurinic/apyrimidinic sites, and base excision-repair.. Prog Nucleic Acid Res Mol Biol.

[OCR_01857] Lindahl T., Nyberg B. (1972). Rate of depurination of native deoxyribonucleic acid.. Biochemistry.

[OCR_01861] Ljungquist S., Andersson A., Lindahl T. (1974). A mammalian endonuclease specific for apurinic sites in double-stranded deoxyribonucleic acid. II. Further studies on the substrate specificity.. J Biol Chem.

[OCR_01866] Loeb L. A. (1985). Apurinic sites as mutagenic intermediates.. Cell.

[OCR_01870] Loeb L. A., Preston B. D. (1986). Mutagenesis by apurinic/apyrimidinic sites.. Annu Rev Genet.

[OCR_01874] Lowy I., Pellicer A., Jackson J. F., Sim G. K., Silverstein S., Axel R. (1980). Isolation of transforming DNA: cloning the hamster aprt gene.. Cell.

[OCR_01879] MacGregor G. R., James M. R., Arlett C. F., Burke J. F. (1987). Analysis of mutations occurring during replication of a SV40 shuttle vector in mammalian cells.. Mutat Res.

[OCR_01884] Malling H. V., De Serres F. J. (1973). Genetic alterations at the molecular level in x-ray induced ad-3B mutants of Neurospora crassa.. Radiat Res.

[OCR_01894] Menck C. F., James M. R., Gentil A., Sarasin A. (1987). Strategies to analyse mutagenesis in mammalian cells using simian virus 40 or shuttle vectors.. J Cell Sci Suppl.

[OCR_01899] Meuth M., Arrand J. E. (1982). Alterations of gene structure in ethyl methane sulfonate-induced mutants of mammalian cells.. Mol Cell Biol.

[OCR_01915] Miller J. H., Low K. B. (1984). Specificity of mutagenesis resulting from the induction of the SOS system in the absence of mutagenic treatment.. Cell.

[OCR_01920] Miller J. K., Barnes W. M. (1986). Colony probing as an alternative to standard sequencing as a means of direct analysis of chromosomal DNA to determine the spectrum of single-base changes in regions of known sequence.. Proc Natl Acad Sci U S A.

[OCR_01927] Mitchell P. J., Urlaub G., Chasin L. (1986). Spontaneous splicing mutations at the dihydrofolate reductase locus in Chinese hamster ovary cells.. Mol Cell Biol.

[OCR_01932] Moore P. D., Song K. Y., Chekuri L., Wallace L., Kucherlapati R. S. (1986). Homologous recombination in a Chinese hamster X-ray-sensitive mutant.. Mutat Res.

[OCR_01937] Moran M. F., Ebisuzaki K. (1987). Base excision repair of DNA in gamma-irradiated human cells.. Carcinogenesis.

[OCR_01941] Muller H. J. (1927). ARTIFICIAL TRANSMUTATION OF THE GENE.. Science.

[OCR_01945] Myers R. M., Larin Z., Maniatis T. (1985). Detection of single base substitutions by ribonuclease cleavage at mismatches in RNA:DNA duplexes.. Science.

[OCR_01950] Nalbantoglu J., Goncalves O., Meuth M. (1983). Structure of mutant alleles at the aprt locus of Chinese hamster ovary cells.. J Mol Biol.

[OCR_01960] Nalbantoglu J., Hartley D., Phear G., Tear G., Meuth M. (1986). Spontaneous deletion formation at the aprt locus of hamster cells: the presence of short sequence homologies and dyad symmetries at deletion termini.. EMBO J.

[OCR_01955] Nalbantoglu J., Phear G. A., Meuth M. (1986). Nucleotide sequence of hamster adenine phosphoribosyl transferase (aprt) gene.. Nucleic Acids Res.

[OCR_01966] Nalbantoglu J., Phear G., Meuth M. (1987). DNA sequence analysis of spontaneous mutations at the aprt locus of hamster cells.. Mol Cell Biol.

[OCR_01971] Novack D. F., Casna N. J., Fischer S. G., Ford J. P. (1986). Detection of single base-pair mismatches in DNA by chemical modification followed by electrophoresis in 15% polyacrylamide gel.. Proc Natl Acad Sci U S A.

[OCR_01977] Olivieri G., Bodycote J., Wolff S. (1984). Adaptive response of human lymphocytes to low concentrations of radioactive thymidine.. Science.

[OCR_01987] Painter R. B., Young B. R. (1987). DNA synthesis in irradiated mammalian cells.. J Cell Sci Suppl.

[OCR_01982] Painter R. B., Young B. R. (1980). Radiosensitivity in ataxia-telangiectasia: a new explanation.. Proc Natl Acad Sci U S A.

[OCR_01991] Paterson M. C., Smith B. P., Lohman P. H., Anderson A. K., Fishman L. (1976). Defective excision repair of gamma-ray-damaged DNA in human (ataxia telangiectasia) fibroblasts.. Nature.

[OCR_01999] Phear G., Nalbantoglu J., Meuth M. (1987). Next-nucleotide effects in mutations driven by DNA precursor pool imbalances at the aprt locus of Chinese hamster ovary cells.. Proc Natl Acad Sci U S A.

[OCR_02005] Ponnamperuma C., Lemmon R. M., Bennett E. L., Calvin M. (1961). Deamination of Adenine by Ionizing Radiation.. Science.

[OCR_02010] Preston R. J. (1982). The use of inhibitors of DNA-repair in the study of the mechanisms of induction of chromosome aberrations.. Cytogenet Cell Genet.

[OCR_02015] Privalle C. T., Fridovich I. (1987). Induction of superoxide dismutase in Escherichia coli by heat shock.. Proc Natl Acad Sci U S A.

[OCR_02020] Razzaque A., Mizusawa H., Seidman M. M. (1983). Rearrangement and mutagenesis of a shuttle vector plasmid after passage in mammalian cells.. Proc Natl Acad Sci U S A.

[OCR_02026] Resnick M. A., Martin P. (1976). The repair of double-strand breaks in the nuclear DNA of Saccharomyces cerevisiae and its genetic control.. Mol Gen Genet.

[OCR_02031] Roginski R. S., Skoultchi A. I., Henthorn P., Smithies O., Hsiung N., Kucherlapati R. (1983). Coordinate modulation of transfected HSV thymidine kinase and human globin genes.. Cell.

[OCR_02037] Rouet P., Essigmann J. M. (1985). Possible role for thymine glycol in the selective inhibition of DNA synthesis on oxidized DNA templates.. Cancer Res.

[OCR_02042] Saiki R. K., Scharf S., Faloona F., Mullis K. B., Horn G. T., Erlich H. A., Arnheim N. (1985). Enzymatic amplification of beta-globin genomic sequences and restriction site analysis for diagnosis of sickle cell anemia.. Science.

[OCR_02052] Schaaper R. M., Danforth B. N., Glickman B. W. (1986). Mechanisms of spontaneous mutagenesis: an analysis of the spectrum of spontaneous mutation in the Escherichia coli lacI gene.. J Mol Biol.

[OCR_02047] Schaaper R. M., Kunkel T. A., Loeb L. A. (1983). Infidelity of DNA synthesis associated with bypass of apurinic sites.. Proc Natl Acad Sci U S A.

[OCR_02058] Seeberg E., Steinum A. L. (1980). Repair of x-ray-induced deoxyribonucleic acid single-strand breaks in xth mutants of Escherichia coli.. J Bacteriol.

[OCR_02063] Shadley J. D., Wolff S. (1987). Very low doses of X-rays can cause human lymphocytes to become less susceptible to ionizing radiation.. Mutagenesis.

[OCR_02079] Shiloh Y., Tabor E., Becker Y. (1983). Abnormal response of ataxia-telangiectasia cells to agents that break the deoxyribose moiety of DNA via a targeted free radical mechanism.. Carcinogenesis.

[OCR_02085] Simic M. G., Dizdaroglu M. (1985). Formation of radiation-induced cross-links between thymine and tyrosine: possible model for cross-linking of DNA and proteins by ionizing radiation.. Biochemistry.

[OCR_02091] Skulimowski A. W., Turner D. R., Morley A. A., Sanderson B. J., Haliandros M. (1986). Molecular basis of X-ray-induced mutation at the HPRT locus in human lymphocytes.. Mutat Res.

[OCR_02097] Smith C. A. (1987). DNA repair in specific sequences in mammalian cells.. J Cell Sci Suppl.

[OCR_02105] Sognier M. A., Hittelman W. N. (1983). Loss of repairability of DNA interstrand crosslinks in Fanconi's anemia cells with culture age.. Mutat Res.

[OCR_02110] Stankowski L. F., Hsie A. W. (1986). Quantitative and molecular analyses of radiation-induced mutation in AS52 cells.. Radiat Res.

[OCR_02115] Strauss B., Rabkin S., Sagher D., Moore P. (1982). The role of DNA polymerase in base substitution mutagenesis on non-instructional templates.. Biochimie.

[OCR_02120] Szostak J. W., Orr-Weaver T. L., Rothstein R. J., Stahl F. W. (1983). The double-strand-break repair model for recombination.. Cell.

[OCR_02101] Söderhäll S., Lindahl T. (1976). DNA ligases of eukaryotes.. FEBS Lett.

[OCR_02125] Takeshita M., Chang C. N., Johnson F., Will S., Grollman A. P. (1987). Oligodeoxynucleotides containing synthetic abasic sites. Model substrates for DNA polymerases and apurinic/apyrimidinic endonucleases.. J Biol Chem.

[OCR_02132] Tan K. H., Meyer D. J., Coles B., Ketterer B. (1986). Thymine hydroperoxide, a substrate for rat Se-dependent glutathione peroxidase and glutathione transferase isoenzymes.. FEBS Lett.

[OCR_02138] Teebor G. W., Duker N. J. (1975). Human endonuclease activity for DNA apurinic sites.. Nature.

[OCR_02142] Teebor G. W., Frenkel K., Goldstein M. S. (1984). Ionizing radiation and tritium transmutation both cause formation of 5-hydroxymethyl-2'-deoxyuridine in cellular DNA.. Proc Natl Acad Sci U S A.

[OCR_02154] Teoule R., Bert C., Bonicel A. (1977). Thymine fragment damage retained in the DNA polynucleotide chain after gamma irradiation in aerated solutions. II.. Radiat Res.

[OCR_02148] Teoule R., Cadet J. (1978). Radiation-induced degradation of the base component in DNA and related substances--final products.. Mol Biol Biochem Biophys.

[OCR_02175] Thacker J., Cox R. (1975). Mutation induction and inactivation in mammalian cells exposed to ionising radiation.. Nature.

[OCR_02187] Thacker J., Stephens M. A., Stretch A. (1978). Mutation to ouabain-resistance in Chinese hamster cells: induction by ethyl methanesulphonate and lack of induction by ionising radiation.. Mutat Res.

[OCR_02159] Thacker J. (1985). The molecular nature of mutations in cultured mammalian cells: a review.. Mutat Res.

[OCR_02168] Thacker J. (1986). The nature of mutants induced by ionising radiation in cultured hamster cells. III. Molecular characterization of HPRT-deficient mutants induced by gamma-rays or alpha-particles showing that the majority have deletions of all or part of the hprt gene.. Mutat Res.

[OCR_02163] Thacker J. (1986). The use of recombinant DNA techniques to study radiation-induced damage, repair and genetic change in mammalian cells.. Int J Radiat Biol Relat Stud Phys Chem Med.

[OCR_02193] Thomas D. C., Kunkel T. A., Casna N. J., Ford J. P., Sancar A. (1986). Activities and incision patterns of ABC excinuclease on modified DNA containing single-base mismatches and extrahelical bases.. J Biol Chem.

[OCR_02204] Totter J. R. (1980). Spontaneous cancer and its possible relationship to oxygen metabolism.. Proc Natl Acad Sci U S A.

[OCR_02220] Urlaub G., Käs E., Carothers A. M., Chasin L. A. (1983). Deletion of the diploid dihydrofolate reductase locus from cultured mammalian cells.. Cell.

[OCR_02209] Urlaub G., Mitchell P. J., Kas E., Chasin L. A., Funanage V. L., Myoda T. T., Hamlin J. (1986). Effect of gamma rays at the dihydrofolate reductase locus: deletions and inversions.. Somat Cell Mol Genet.

[OCR_02231] Vrieling H., Simons J. W., Arwert F., Natarajan A. T., van Zeeland A. A. (1985). Mutations induced by X-rays at the HPRT locus in cultured Chinese hamster cells are mostly large deletions.. Mutat Res.

[OCR_02237] Wake C. T., Wilson J. H. (1979). Simian virus 40 recombinants are produced at high frequency during infection with genetically mixed oligomeric DNA.. Proc Natl Acad Sci U S A.

[OCR_02242] Walker G. C. (1984). Mutagenesis and inducible responses to deoxyribonucleic acid damage in Escherichia coli.. Microbiol Rev.

[OCR_02246] Ward J. F. (1985). Biochemistry of DNA lesions.. Radiat Res Suppl.

[OCR_02250] Ward J. F., Kuo I. (1976). Strand breaks, base release, and postirradiation changes in DNA gamma-irradiated in dilute O2-saturated aqueous solution.. Radiat Res.

[OCR_02255] Weinert T. A., Derbyshire K. M., Hughson F. M., Grindley N. D. (1984). Replicative and conservative transpositional recombination of insertion sequences.. Cold Spring Harb Symp Quant Biol.

[OCR_02263] Willis A. E., Lindahl T. (1987). DNA ligase I deficiency in Bloom's syndrome.. Nature.

[OCR_02267] Wilson J. M., Stout J. T., Palella T. D., Davidson B. L., Kelley W. N., Caskey C. T. (1986). A molecular survey of hypoxanthine-guanine phosphoribosyltransferase deficiency in man.. J Clin Invest.

[OCR_02273] Witkin E. M. (1976). Ultraviolet mutagenesis and inducible DNA repair in Escherichia coli.. Bacteriol Rev.

[OCR_02277] Wood R. D., Burki H. J., Hughes M., Poley A. (1983). Radiation-induced lethality and mutation in a repair-deficient CHO cell line.. Int J Radiat Biol Relat Stud Phys Chem Med.

[OCR_02282] Wood W. I., Gitschier J., Lasky L. A., Lawn R. M. (1985). Base composition-independent hybridization in tetramethylammonium chloride: a method for oligonucleotide screening of highly complex gene libraries.. Proc Natl Acad Sci U S A.

[OCR_02289] Yandell D. W., Dryja T. P., Little J. B. (1986). Somatic mutations at a heterozygous autosomal locus in human cells occur more frequently by allele loss than by intragenic structural alterations.. Somat Cell Mol Genet.

[OCR_02300] Yang T. P., Patel P. I., Chinault A. C., Stout J. T., Jackson L. G., Hildebrand B. M., Caskey C. T. (1984). Molecular evidence for new mutation at the hprt locus in Lesch-Nyhan patients.. Nature.

[OCR_02305] Yatagai F., Horsfall M. J., Glickman B. W. (1987). Defect in excision repair alters the mutational specificity of PUVA treatment in the lacI gene of Escherichia coli.. J Mol Biol.

[OCR_01451] de Saint Vincent B. R., Wahl G. M. (1983). Homologous recombination in mammalian cells mediates formation of a functional gene from two overlapping gene fragments.. Proc Natl Acad Sci U S A.

